# Updated hoverfly (Diptera, Syrphidae) checklist of the Foreste Casentinesi National Park (Italy)

**DOI:** 10.3897/BDJ.13.e147052

**Published:** 2025-05-29

**Authors:** Giovanni Burgio, Martina Di Saverio, Guido Crudele, Daniele Sommaggio

**Affiliations:** 1 Dipartimento di Scienze e Tecnologie Agro‑Alimentari, Alma Mater Studiorum Università di Bologna, Bologna, Italy Dipartimento di Scienze e Tecnologie Agro‑Alimentari, Alma Mater Studiorum Università di Bologna Bologna Italy; 2 Freelance, Padova, Italy Freelance Padova Italy; 3 Ufficio Territoriale per la biodiversità, Corpo Forestale dello Stato, Pratovecchio, AR, Italy Ufficio Territoriale per la biodiversità, Corpo Forestale dello Stato Pratovecchio, AR Italy; 4 Department of Life Sciences, University of Modena and Reggio Emilia, Reggio Emilia, Italy Department of Life Sciences, University of Modena and Reggio Emilia Reggio Emilia Italy; 5 National Biodiversity Future Center, Palermo, Italy National Biodiversity Future Center Palermo Italy

**Keywords:** Syrphidae (Diptera), Italian fauna, National Park checklist, Lama forest, biodiversity, nature conservation

## Abstract

**Background:**

The present faunistic note is an updating of Syrphidae (Diptera) fauna of “Foreste Casentinesi National Park” (Emilia-Romagna and Toscana, Italy), a site of conservation interest included as a focus site within the “National Biodiversity Future Center”, NBFC. The revision combines data from the entomological collection Guido Grandi (UNIBO) and recent records from the national pollinator monitoring programme (Italy).

**New information:**

The new recordings for the National Park, obtained by Malaise traps and entomological net, comprise 25 Syrphidae species, including also new recordings for Toscana and Emilia-Romagna Regions. The updated Syrphidae fauna of the Park includes now 116 species, three of which are included within threatened categories in the Red List of European Syrphidae. A typically Alpine fauna is emerging from the Syrphidae species recorded of this revision.

## Introduction

The foundation for any action in biodiversity conservation is to have inventories of specific taxa, based on literature and database updating. By providing information on the place and time of collection, specimens can be used to generate spatio-temporal data on the geographical distribution, phenology and other phenotypic traits of organisms (Kharouba et al. 2019). The enormous potential of natural history collection data for examining insect responses at temporal and spatial scales has been demonstrated, corroborating the importance of biological collections for understanding biodiversity in the Anthropocene (Meier and Dikov 2004, Meineke et al. 2018, Kharouba et al. 2019, Sánchez-Fernández et al. 2022). Dynamic processes of communities and ecosystems across different protection levels can be investigated by using an historical ecology perspective, thus reconstructing biodiversity and ecosystem changes over a timescale from decades to centuries. Biodiversity decline and climate change are considered important emergencies in the current era, thus promoting global interests aimed at conservation actions (Pörtner et al. 2021).

Hoverflies (Diptera, Syrphidae) are an important component of the flower-visiting insect fauna (Rader et al. 2020, Doyle et al. 2020) and have recently been included as a taxonomic group for the EU Pollinator Monitoring Scheme (Potts et al. 2021). Besides the role as pollinators, hoverflies provide other ecosystem services, such as biological control (Rodríguez-Gasol et al. 2020, Pekas et al. 2020, Burgio et al. 2024) and are considered effective bioindicators of natural and anthropogenised environments (Sommaggio 1999), whose use is facilitated by the development of the expert system Syrph the Net (StN) (Speight and Castella 2001). It should be emphasised that updated regional hoverfly lists are required to apply StN and provide effective conservation strategies (Speight 2012). Italy is the second richest country in Europe considering the number of hoverfly species (Reverté et al. 2023); however, little is known about the distribution of hoverflies in Italian regions; some areas, especially in central and southern Italy, are particularly poor in known hoverfly species, indicating a possible underestimation (Sommaggio and Burgio 2004, Sommaggio 2005). In Italy, following previous European initiatives (Potts et al. 2021), a national pollinator monitoring programme has been promoted within national parks, to assess the conservation status of pollinators, including hoverflies (D'Antoni et al. 2020). In light of the above, hoverflies certainly have the characteristics to be considered a fundamental taxon for biodiversity surveillance and ecological habitat assesments. Updating faunistic lists of sites of conservation importance is crucial for the spatial/temporal evaluation of biodiversity trends and maintain or reinforce conservation measures (Cazzolla Gatti et al. 2023).

The aim of this work is to update the hoverfly fauna of Foreste Casentinesi, Monte Falterona and Campigna National Park (FCNP), an important Italian park of conservation interest, by a revision of material collected over the years and never published. The only published recordings of hoverflies of FCNP are available in Burgio et al. (2000). These historical data have been integrated with new records recently collected from FCNP through the national pollinator monitoring programme, generating a significative revision of the hoverfly fauna.

## Materials and methods

The FCNP was established in 1993 and covers an area of 368 km^2^, spanning the regions of Emilia Romagna and Toscana. The parks extends over an altitude range of 500 – 1660 m and can therefore be considered mountainous. Forests are the dominant habitat; more than one quarter of all FCNP is covered by beech forest, some of them of great conservation interest, such as Sasso Fratino Forest included in 2017 in the World Heritage Site by the UNESCO Commission (Viciani and Agostini 2009, Viciani and Alberti 2024). From a climatic point of view the area belongs to the temperate oceanic bioclimate and marginally, at lower altitude, to the temperate sub-Mediterranean bioclimate (Padula and Crudele 1988, Pesaresi et al. 2017). This Park has been involved in the national pollinator monitoring programme (D'Antoni et al. 2020), established by Ministero dell’Ambiente e della Sicurezza Energetica and Istituto Superiore per la Protezione e la Ricerca Ambientale. Moreover, FCNP is also included as a focus site within the “National Biodiversity Future Center”, NBFC.

**Figure 1.**
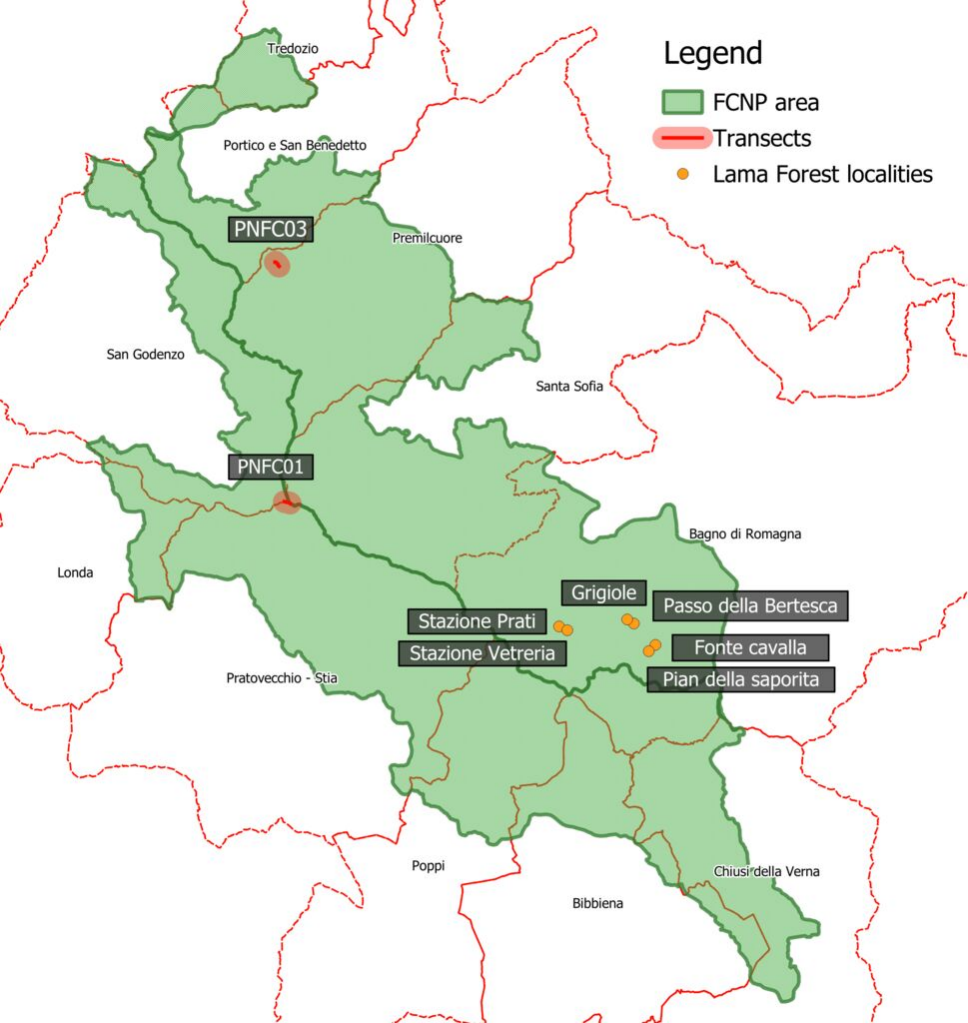
FCNP map and the positions of Malaise traps in 2003/04 (in orange) and permanent transect sampled in 2023 (in red).

The present inventory updating is mostly based on a revision of hoverfly specimens deposited in the “Guido Grandi Collection” (University of Bologna) (Sommaggio 2010) and previously collected within FCNP. Most of the material (354 specimens) deposited in the “Guido Grandi Collection” was collected between 2003 and 2004 using Malaise traps. The traps were set from the first week of May to mid-August in 2003 and from the second week of May to end of July in 2004. Malaise traps were located in “La Lama Forest” (700-1275 m) (Fig. 1). In particular, the following localities were sampled: Fonte Cavalla, 1050 m; Pian della Saporita, 1051 m; Passo della Bertesca, 1275 m; Grigiole, 1003 m; Stazione Vetreria, 720 m; Stazione Prati (Casa Forestale Lama), 700 m (Fig. 1). The study was carried out with the permission and the contribution of the “Ufficio Territoriale per la biodiversità” (Corpo Forestale dello Stato, Pratovacchio, AR Province). A smaller part of the material deposited in “Guido Grandi Collection” (24 hoverfly specimens) was hand-collected between 1984 and 1997 by Guido Campadelli. All specimens are now preserved in the "Guido Grandi Collection", University of Bologna (acronym AMSUB).

The more recent material was collected in 2023 in FCNP with an entomological net in two transects (250 m long), located in Monte Falco (average altitude of 1653 m) and Bucine (average altitude of 907 m) (Fig. 1). Monte Falco is in the section of the ridge between Toscana and Romagna where almost all the high-altitude meadows of the Park are concentrated. They consist of habitat 6230 of the Rete Natura 2000, with co-occurrence of habitat 4060, by the presence of *Vaccinium* spp. These open areas are surrounded by predominantly pure beech forests (habitat 9130). Bucine transect consists of a mixed forest of oak and hornbeam for about the first quarter of its path and the rest of the path is characterised by a continuum from mixed to pure beech forest, from lower to higher altitude. Then the transect continues in semi-natural pastures with the presence of shrubs (habitat 6210 in prevalence) and also some fruit trees, such as cherries and pears. The applied sampling method followed the protocol of the national pollinator monitoring programme to create baseline data for the evaluation of pollinator trends (D'Antoni et al. 2020). Two transects were selected to study the bee and hoverfly communities: each transect was monitored on one day each month, with surveys conducted every two hours throughout the day. The complete results of this research will be the object of another paper; here, preliminary data have been used to integrate the fauna of FCNP. Transects were visited from May to September 2023; each one was walked one day a month, every two hours from 9 am to 5 pm and they took around 45 minutes to complete. All hoverflies detected along the transect were identified in the field or collected for subsequent identification. Specimens collected and identified are preserved in the collection of FCNP identified with the label PNFCC (Collection of the Foreste Casentinesi, Monte Falterona and Campigna National Park, deposited in Santa Sofia, province of FC).

## Checklists

### Uploaded checklist of PNFC Syrphidae

#### Baccha elongata

(Fabricius, 1775)

E37E7368-F09C-54BB-9A3A-C60ED7461A2D

##### Materials

- **Type status:** Other material. **Occurrence:** sex: 1 male; occurrenceID: 9B2DAE95-831F-50E3-A0D3-45EAF70CA82D; **Taxon:** scientificName: Bacchaelongata (Fabricius, 1775); order: Diptera; family: Syrphidae; **Location:** country: Italy; locality: PNFC Foresta Lama Bertesca (FC); decimalLatitude: 43.8314943; decimalLongitude: 11.8735824; geodeticDatum: WGS84; **Event:** samplingProtocol: Malaise Trap; verbatimEventDate: 27.vii.2004; **Record Level:** collectionID: AMSUB005151
- **Type status:** Other material. **Occurrence:** sex: 1 female; occurrenceID: AC330CAF-CEC2-5E09-AD90-31F5EAD96D0E; **Taxon:** scientificName: Bacchaelongata (Fabricius, 1775); order: Diptera; family: Syrphidae; **Location:** country: Italy; locality: PNFC Foresta Lama Vetreria (FC); decimalLatitude: 43.829961; decimalLongitude: 11.8380049; geodeticDatum: WGS84; **Event:** samplingProtocol: Malaise Trap; verbatimEventDate: 3-14.vii.2003; **Record Level:** collectionID: AMSUB005326
- **Type status:** Other material. **Occurrence:** occurrenceID: A92445F1-C70C-5BF7-B468-20640C71AD29; **Taxon:** scientificName: Bacchaelongata (Fabricius, 1775); order: Diptera; family: Syrphidae; **Location:** country: Italy; locality: PNFC Bucine (FC); decimalLatitude: 43.9594; decimalLongitude: 11.7097; geodeticDatum: WGS84; **Event:** samplingProtocol: Observation; verbatimEventDate: 12.vi.2023

##### Notes

Recorded in Burgio et al. (2000).

#### Brachyopa pilosa

Collin, 1939

C88DEC6B-7158-54F1-BDD3-2DED679380F1

##### Notes

Recorded in Burgio et al. (2000).

#### Brachypalpoides lentus

(Meigen, 1822)

A8C2C789-0096-5E7F-A8EE-C7966F5FA8F7

##### Notes

Recorded in Burgio et al. (2000).

#### Brachypalpus laphriformis

(Fallén, 1816)

37DF1E7E-0CF2-5B0E-AF85-0C2780D1ABB5

##### Notes

Recorded in Burgio et al. (2000).

#### Caliprobola speciosa

(Rossi, 1790)

80E2683A-D4D6-5B68-85CA-11C86AA92613

##### Notes

Recorded in Burgio et al. (2000).

#### Callicera aurata

(Rossi, 1790)

9181EE6E-B267-51F7-824A-25E745CFF8E0

##### Materials

- **Type status:** Other material. **Occurrence:** sex: 2 females; occurrenceID: 803C8A56-BD12-5329-9CB4-B15C74B97322; **Taxon:** scientificName: Calliceraaurata (Rossi, 1790); order: Diptera; family: Syrphidae; **Location:** country: Italy; locality: PNFC Bucine (FC); decimalLatitude: 43.9594; decimalLongitude: 11.7097; geodeticDatum: WGS84; **Event:** samplingProtocol: Entomological Net; verbatimEventDate: 12.viii.2023; **Record Level:** collectionID: PNFCC000002, PNFCC000003

#### Cheilosia barbata

Loew, 1857

89A3646E-A786-5B32-B9D6-8CBC64668F9E

##### Materials

- **Type status:** Other material. **Occurrence:** sex: 1 female; occurrenceID: 0802E1A4-AE0E-5BA2-8861-72739F940611; **Taxon:** scientificName: Cheilosiabarbata Loew, 1857; order: Diptera; family: Syrphidae; **Location:** country: Italy; locality: PNFC Le Culacce (FC); decimalLatitude: 43.8628; decimalLongitude: 11.7527; geodeticDatum: WGS84; **Event:** samplingProtocol: Malaise Trap; verbatimEventDate: 2.viii.1997; **Record Level:** collectionID: AMSUB005331
- **Type status:** Other material. **Occurrence:** sex: 1 female; occurrenceID: AA1FFC89-5754-531B-A8D3-78FA72F1D1F7; **Taxon:** scientificName: Cheilosiabarbata Loew, 1857; order: Diptera; family: Syrphidae; **Location:** country: Italy; locality: PNFC Le Culacce (FC); decimalLatitude: 43.8628; decimalLongitude: 11.7527; geodeticDatum: WGS84; **Event:** samplingProtocol: Malaise Trap; verbatimEventDate: 27.vii.1996; **Record Level:** collectionID: AMSUB005332
- **Type status:** Other material. **Occurrence:** sex: 1 male; occurrenceID: 72694EAB-A469-5474-BEA2-49654ECE05D2; **Taxon:** scientificName: Cheilosiabarbata Loew, 1857; order: Diptera; family: Syrphidae; **Location:** country: Italy; locality: PNFC Le Culacce (FC); decimalLatitude: 43.8628; decimalLongitude: 11.7527; geodeticDatum: WGS84; **Event:** samplingProtocol: Malaise Trap; verbatimEventDate: 18.vi.1994; **Record Level:** collectionID: AMSUB005336
- **Type status:** Other material. **Occurrence:** sex: 1 female; occurrenceID: 1998C30D-B050-5FA2-97F2-B1EBFD0D5447; **Taxon:** scientificName: Cheilosiabarbata Loew, 1857; order: Diptera; family: Syrphidae; **Location:** country: Italy; locality: PNFC Campigna (FC); decimalLatitude: 43.8328; decimalLongitude: 11.8114; geodeticDatum: WGS84; **Event:** samplingProtocol: Entomological Net; verbatimEventDate: 15.vii.1994; **Record Level:** collectionID: AMSUB005338

##### Notes

Recorded in Burgio et al. (2000).

#### Cheilosia bracusi

Vujić & Claussen, 1994

8572DF32-4CD2-5387-BDCA-46BC7D842927

##### Notes

Recorded in Burgio et al. (2000).

#### Cheilosia canicularis

(Panzer, 1801)

960DABA7-7943-5926-B49A-2B0F6744CBE3

##### Materials

- **Type status:** Other material. **Occurrence:** sex: 1 male, 1 female; occurrenceID: 926F468F-9FCF-563D-BED6-9C7EC252415B; **Taxon:** scientificName: Cheilosiacanicularis (Panzer, 1801); order: Diptera; family: Syrphidae; **Location:** country: Italy; locality: PNFC Piedimonte (FI); decimalLatitude: 44.0924; decimalLongitude: 11.5185; geodeticDatum: WGS84; **Event:** samplingProtocol: Entomological Net; verbatimEventDate: 20.viii.1994; **Record Level:** collectionID: AMSUB005333, AMSUB005335
- **Type status:** Other material. **Occurrence:** sex: 1 male; occurrenceID: D29A2784-299D-5679-9DB2-79B7C440D50C; **Taxon:** scientificName: Cheilosiacanicularis (Panzer, 1801); order: Diptera; family: Syrphidae; **Location:** country: Italy; locality: PNFC La Stretta (FC); **Event:** samplingProtocol: Entomological Net; verbatimEventDate: 18.vii.1993; **Record Level:** collectionID: AMSUB005339

##### Notes

Recorded in Burgio et al. (2000).

#### Cheilosia carbonaria

Egger, 1860

C622DE11-6E5F-5CEB-A88A-5D37C7BBCB28

##### Notes

Recorded in Burgio et al. (2000).

#### Cheilosia himantopus

(Panzer, 1798)

6EFEE9E3-30A0-5816-8336-7E66DE1211E0

##### Notes

Recorded in Burgio et al. (2000).

#### Cheilosia impressa

Loew, 1840

46A772F9-2F48-5C59-8B4D-06AE2C6F5F13

##### Materials

- **Type status:** Other material. **Occurrence:** sex: 1 male; occurrenceID: 9BB099A0-A0E3-504B-B045-56177B5F228B; **Taxon:** scientificName: Cheilosiaimpressa Loew, 1840; order: Diptera; family: Syrphidae; **Location:** country: Italy; locality: PNFC Palazzuolo sul Senio (FI); decimalLatitude: 44.1135; decimalLongitude: 11.5468; geodeticDatum: WGS84; **Event:** samplingProtocol: Entomological Net; verbatimEventDate: viii.85; **Record Level:** collectionID: AMSUB005337
- **Type status:** Other material. **Occurrence:** sex: 1 female; occurrenceID: 5E1779CE-4061-55B5-8EAF-60A1986931BB; **Taxon:** scientificName: Cheilosiaimpressa Loew, 1840; order: Diptera; family: Syrphidae; **Location:** country: Italy; locality: PNFC Bucine (FC); decimalLatitude: 43.9594; decimalLongitude: 11.7097; geodeticDatum: WGS84; **Event:** samplingProtocol: Entomological Net; verbatimEventDate: 12.vi.2023; **Record Level:** collectionID: PNFCC000004

##### Notes

Recorded in Burgio et al. (2000).

#### Cheilosia melanura

Becker, 1894

EE030026-11EB-59BD-8ADD-B049504F1A6D

##### Materials

- **Type status:** Other material. **Occurrence:** sex: 1 male; occurrenceID: 368BF39A-37D1-5B82-807E-7CAD8EFB594B; **Taxon:** scientificName: Cheilosiamelanura Becker, 1894; order: Diptera; family: Syrphidae; **Location:** country: Italy; locality: PNFC Monte Falco (FI); decimalLatitude: 43.8773; decimalLongitude: 11.7107; geodeticDatum: WGS84; **Event:** samplingProtocol: Entomological Net; verbatimEventDate: 6.v.2023; **Record Level:** collectionID: PNFCC000005

#### Cheilosia mutabilis

(Fallén, 1817)

277A2387-E3FC-5357-8B95-4A358C39106E

##### Notes

Recorded in Burgio et al. (2000).

#### Cheilosia nigripes

(Meigen, 1822)

543419D2-E60E-5D8B-8FFE-71B50E4891B4

##### Materials

- **Type status:** Other material. **Occurrence:** sex: 1 female; occurrenceID: 47F44189-7A2D-50E9-AA6D-3B7F7E6E848A; **Taxon:** scientificName: Cheilosianigripes (Meigen, 1822); order: Diptera; family: Syrphidae; **Location:** country: Italy; locality: PNFC Foresta Lama Stazione Prati (FC); decimalLatitude: 43.8313707; decimalLongitude: 11.8380049; geodeticDatum: WGS84; **Event:** samplingProtocol: Malaise Trap; verbatimEventDate: 26.v-6.vi.2003; **Record Level:** collectionID: AMSUB005193

##### Notes

Recorded in Burgio et al. (2000).

#### Cheilosia proxima

(Zetterstedt), 1843

AAD23097-5B09-56C0-A63F-4FC493313E6B

##### Materials

- **Type status:** Other material. **Occurrence:** sex: 1 female; occurrenceID: DE84A94F-61FC-5F03-9B9E-CB0C714D9818; **Taxon:** scientificName: Cheilosiaproxima (Zetterstedt), 1843; order: Diptera; family: Syrphidae; **Location:** country: Italy; locality: PNFC Monte Falco (FI) 1653m; decimalLatitude: 43.8773; decimalLongitude: 11.7107; geodeticDatum: WGS84; **Event:** samplingProtocol: Entomological Net; verbatimEventDate: 11.vi.2023; **Record Level:** collectionID: PNFCC000006

#### Cheilosia ranunculi

Doczkal, 2000

F573D213-CD56-59C8-8B5A-9083AAA402A2

##### Materials

- **Type status:** Other material. **Occurrence:** sex: 1 female; occurrenceID: A5446CAE-905A-5FE6-A538-D3A40248111C; **Taxon:** scientificName: Cheilosiaranunculi Doczkal, 2000; order: Diptera; family: Syrphidae; **Location:** country: Italy; locality: PNFC Foresta Lama Stazione Prati; decimalLatitude: 43.8313707; decimalLongitude: 11.8380049; geodeticDatum: WGS84; **Event:** samplingProtocol: Malaise Trap; verbatimEventDate: 26.v-6.vi.2003; **Record Level:** collectionID: AMSUB005210

#### Cheilosia rhynchops

Egger, 1860

C3FD8ACC-DE0D-546C-A540-D40EAA44E61D

##### Materials

- **Type status:** Other material. **Occurrence:** sex: 1 female; occurrenceID: C0925109-10A5-5696-ABF5-95E74683B9C0; **Taxon:** scientificName: Cheilosiarhynchops Egger, 1860; order: Diptera; family: Syrphidae; **Location:** country: Italy; locality: PNFC Le Cullacce Campigna m. 1041; decimalLatitude: 43.8628; decimalLongitude: 11.7527; geodeticDatum: WGS84; **Event:** samplingProtocol: Entomological Net; verbatimEventDate: 18.vi.1994; **Record Level:** collectionID: AMSUB005334

##### Notes

Recorded in Burgio et al. (2000).

#### Cheilosia scutellata

(Fallén, 1817)

731969E0-E652-579E-B7B4-3A9D2378E79A

##### Notes

Recorded in Burgio et al. (2000).

#### Cheilosia variabilis

(Panzer, 1798)

AF445ED5-F6FC-54EE-9544-76FB438986E1

##### Materials

- **Type status:** Other material. **Occurrence:** sex: 1 female; occurrenceID: 022010EE-2B24-55AC-8097-B5A48F9A6218; **Taxon:** scientificName: Cheilosiavariabilis (Panzer, 1798); order: Diptera; family: Syrphidae; **Location:** country: Italy; locality: PNFC Monte Falco (FI) 1653m; decimalLatitude: 43.8773; decimalLongitude: 11.7107; geodeticDatum: WGS84; **Event:** samplingProtocol: Entomological Net; verbatimEventDate: 11.vi.2023; **Record Level:** collectionID: PNFCC000007
- **Type status:** Other material. **Occurrence:** sex: 1 female; occurrenceID: 63AC05CA-FD15-5988-A4DB-C58E5DA54081; **Taxon:** scientificName: Cheilosiavariabilis (Panzer, 1798); order: Diptera; family: Syrphidae; **Location:** country: Italy; locality: PNFC Bucine (FC); decimalLatitude: 43.9594; decimalLongitude: 11.7097; geodeticDatum: WGS84; **Event:** samplingProtocol: Entomological Net; verbatimEventDate: 12.vi.2023; **Record Level:** collectionID: PNFCC000008

##### Notes

Recorded in Burgio et al. (2000).

#### Chrysogaster solstitialis

(Fallén, 1817)

C8277371-C8EC-57F0-A826-F1F3FF812FE3

##### Notes

Recorded in Burgio et al. (2000).

#### Chrysotoxum arcuatum

(Linnaeus, 1758)

DDA27AAE-A69C-5F48-9BBD-DDA7A6200FD6

##### Notes

Recorded in Burgio et al. (2000).

#### Chrysotoxum bicinctum

(Linnaeus, 1758)

DBE72A98-34AB-5BA9-8301-165226EBA0DA

##### Notes

Recorded in Burgio et al. (2000).

#### Chrysotoxum cautum

(Harris, 1776)

38F062C0-A04B-5426-8F86-DE1A89747033

##### Notes

Recorded in Burgio et al. (2000).

#### Chrysotoxum fasciolatum

(De Geer, 1776)

AAD27054-77C8-57FF-A94C-A36571B89953

##### Materials

- **Type status:** Other material. **Occurrence:** sex: 1 male; occurrenceID: 43F523F0-6CDE-5A1F-AF05-BECD049B75F1; **Taxon:** scientificName: Chrysotoxumfasciolatum (De Geer, 1776); order: Diptera; family: Syrphidae; **Location:** country: Italy; locality: PNFC Foresta Lama Fonte Cavalla; decimalLatitude: 43.8238539; decimalLongitude: 11.8835054; geodeticDatum: WGS84; **Event:** samplingProtocol: Malaise Trap; verbatimEventDate: 18.v-8.vi.2004; **Record Level:** collectionID: AMSUB005022
- **Type status:** Other material. **Occurrence:** sex: 1 male; occurrenceID: A75580F7-27CF-55A2-A82F-0AC386C7B259; **Taxon:** scientificName: Chrysotoxumfasciolatum (De Geer, 1776); order: Diptera; family: Syrphidae; **Location:** country: Italy; locality: PNFC Foresta Lama Bertesca; decimalLatitude: 43.8314943; decimalLongitude: 11.8735824; geodeticDatum: WGS84; **Event:** samplingProtocol: Malaise Trap; verbatimEventDate: 30.vi.2004; **Record Level:** collectionID: AMSUB005141
- **Type status:** Other material. **Occurrence:** sex: 1 male; occurrenceID: 92F2A9A7-C623-5534-BBFB-922A82325F72; **Taxon:** scientificName: Chrysotoxumfasciolatum (De Geer, 1776); order: Diptera; family: Syrphidae; **Location:** country: Italy; locality: PNFC Foresta Lama Bertesca; decimalLatitude: 43.8314943; decimalLongitude: 11.8735824; geodeticDatum: WGS84; **Event:** samplingProtocol: Malaise Trap; verbatimEventDate: 27.vii.2004; **Record Level:** collectionID: AMSUB005152

##### Notes

Recorded in Burgio et al. (2000).

#### Chrysotoxum festivum

(Linnaeus, 1758)

DC40E519-5490-5DE4-B9EA-15EFBCAFB356

##### Notes

Recorded in Burgio et al. (2000).

#### Chrysotoxum intermedium

Meigen, 1822

BBBE592E-96C9-5BEC-9047-FBC87376107D

##### Materials

- **Type status:** Other material. **Occurrence:** sex: 1 male; occurrenceID: 5E86A5C0-02EB-5E49-8B79-994436B8AECA; **Taxon:** scientificName: Chrysotoxumintermedium Meigen, 1822; order: Diptera; family: Syrphidae; **Location:** country: Italy; locality: PNFC Foresta Lama Fonte Cavalla; decimalLatitude: 43.8238539; decimalLongitude: 11.8835054; geodeticDatum: WGS84; **Event:** samplingProtocol: Malaise Trap; verbatimEventDate: 18.v-8.vi.2004; **Record Level:** collectionID: AMSUB005023
- **Type status:** Other material. **Occurrence:** sex: 1 female; occurrenceID: 095104DF-FACE-5119-9354-668D788A70E0; **Taxon:** scientificName: Chrysotoxumintermedium Meigen, 1822; order: Diptera; family: Syrphidae; **Location:** country: Italy; locality: PNFC Foresta Lama P. Saporita; decimalLatitude: 43.821774; decimalLongitude: 11.8802281; geodeticDatum: WGS84; **Event:** samplingProtocol: Malaise Trap; verbatimEventDate: 27.vii.2004; **Record Level:** collectionID: AMSUB005027
- **Type status:** Other material. **Occurrence:** sex: 1 male, 3 females; occurrenceID: CC742CC5-5345-56ED-9727-F6370287EE57; **Taxon:** scientificName: Chrysotoxumintermedium Meigen, 1822; order: Diptera; family: Syrphidae; **Location:** country: Italy; locality: PNFC Foresta Lama P. Saporita; decimalLatitude: 43.821774; decimalLongitude: 11.8802281; geodeticDatum: WGS84; **Event:** samplingProtocol: Malaise Trap; verbatimEventDate: 9.vii.2004; **Record Level:** collectionID: AMSUB005061, AMSUB005062, AMSUB005063, AMSUB005064

#### Chrysotoxum lessonae

(Giglio-Tos, 1890)

00043132-FFA6-5952-8C62-7C55458ECB9F

##### Notes

Recorded in Burgio et al. (2000).

#### Chrysotoxum octomaculatum

Curtis, 1837

BC4708F4-05D3-5D37-9DCC-8BC37B93FFD3

##### Materials

- **Type status:** Other material. **Occurrence:** sex: 1 female; occurrenceID: 63ADE0B3-F21C-58C2-938B-D94B0C538C53; **Taxon:** scientificName: Chrysotoxumoctomaculatum Curtis, 1837; order: Diptera; family: Syrphidae; **Location:** country: Italy; locality: PNFC Foresta Lama Bertesca; decimalLatitude: 43.8314943; decimalLongitude: 11.8735824; geodeticDatum: WGS84; **Event:** samplingProtocol: Malaise Trap; verbatimEventDate: 27.vii.2004; **Record Level:** collectionID: AMSUB005153

#### Chrysotoxum vernale

Loew, 1841

75AEFE0C-F724-5E75-B9AF-731DEBF56698

##### Materials

- **Type status:** Other material. **Occurrence:** sex: 1 female; occurrenceID: BAAEF134-634F-5D59-B98F-360080FAD9F1; **Taxon:** scientificName: Chrysotoxumvernale Loew, 1841; order: Diptera; family: Syrphidae; **Location:** country: Italy; locality: PNFC Foresta Lama Fonte Cavalla; decimalLatitude: 43.8238539; decimalLongitude: 11.8835054; geodeticDatum: WGS84; **Event:** samplingProtocol: Malaise Trap; verbatimEventDate: 18.v-8.vi.2004; **Record Level:** collectionID: AMSUB005024

#### Criorhina asilica

(Fallén, 1816)

D0BF00FA-66AE-581C-B59D-81D8120BEE44

##### Notes

Recorded in Burgio et al. (2000).

#### Criorhina floccosa

(Meigen, 1822)

5A0D1AA9-EC54-53D0-A178-2A0ABFDE9FA8

##### Notes

Recorded in Burgio et al. (2000).

#### Dasysyrphus albostriatus

(Fallén, 1817)

226E14C6-8E17-5F9B-A73B-C6EDAE78038B

##### Materials

- **Type status:** Other material. **Occurrence:** sex: 1 male; occurrenceID: BB74DF5D-52AA-55A2-8406-1321EF2C7C29; **Taxon:** scientificName: Dasysyrphusalbostriatus (Fallén, 1817); order: Diptera; family: Syrphidae; **Location:** country: Italy; locality: PNFC Foresta Lama P. Saporita; decimalLatitude: 43.821774; decimalLongitude: 11.8802281; geodeticDatum: WGS84; **Event:** samplingProtocol: Malaise Trap; verbatimEventDate: 27.vii.2004; **Record Level:** collectionID: AMSUB005126
- **Type status:** Other material. **Occurrence:** sex: 1 female; occurrenceID: 08CA8071-31EF-5D09-BE6D-607536964F34; **Taxon:** scientificName: Dasysyrphusalbostriatus (Fallén, 1817); order: Diptera; family: Syrphidae; **Location:** country: Italy; locality: PNFC Foresta Lama Stazione Prati; decimalLatitude: 43.8313707; decimalLongitude: 11.8380049; geodeticDatum: WGS84; **Event:** samplingProtocol: Malaise Trap; verbatimEventDate: 3-14.vii.2003; **Record Level:** collectionID: AMSUB005283

##### Notes

Recorded in Burgio et al. (2000).

#### Dasysyrphus friuliensis

(van der Goot, 1960)

F5F8730A-3E6F-5341-B3FD-0A97F9638DE2

##### Materials

- **Type status:** Other material. **Occurrence:** sex: 1 female; occurrenceID: 977598ED-51BB-5BC2-9BC3-6A7DECA3FDCF; **Taxon:** scientificName: Dasysyrphusfriuliensis (van der Goot, 1960); order: Diptera; family: Syrphidae; **Location:** country: Italy; locality: PNFC Piedimonte (FI) m. 500; decimalLatitude: 44.0924; decimalLongitude: 11.5185; geodeticDatum: WGS84; **Event:** samplingProtocol: Entomological Net; verbatimEventDate: 15.viii.1994; **Record Level:** collectionID: AMSUB005347

#### Dasysyrphus pinastri

(De Geer, 1776)

6DD72880-B59D-5E07-9621-5D278393F444

##### Notes

Recorded in Burgio et al. (2000).

#### Dasysyrphus tricinctus

(Fallén, 1817)

4C3AAA8C-6522-5E28-A080-00F8749F7C19

##### Notes

Recorded in Burgio et al. (2000).

#### Dasysyrphus venustus

(Meigen), 1822

A4BE52C6-4FDA-5221-BDC9-48CBC9DE44F0

##### Materials

- **Type status:** Other material. **Occurrence:** sex: 1 female; occurrenceID: 9A3739EE-2CE7-51A0-B84E-D7624E06BF9E; **Taxon:** scientificName: Dasysyrphusvenustus (Meigen), 1822; order: Diptera; family: Syrphidae; **Location:** country: Italy; locality: PNFC Monte Falco (FI) 1653m; decimalLatitude: 43.8773; decimalLongitude: 11.7107; geodeticDatum: WGS84; **Event:** samplingProtocol: Entomological Net; verbatimEventDate: 11.vi.2023; **Record Level:** collectionID: PNFCC000009

#### Didea fasciata

Macquart, 1843

E86A9477-657D-5D71-B330-FB510ECB893B

##### Materials

- **Type status:** Other material. **Occurrence:** sex: 1 female; occurrenceID: 96619D56-2A15-58E8-9A57-416D3412DFE5; **Taxon:** scientificName: Dideafasciata Macquart, 1843; order: Diptera; family: Syrphidae; **Location:** country: Italy; locality: PNFC Foresta Lama Grigiole 800 m; decimalLatitude: 43.832961; decimalLongitude: 11.8704871; geodeticDatum: WGS84; **Event:** samplingProtocol: Malaise Trap; verbatimEventDate: 6.viii.2003; **Record Level:** collectionID: AMSUB005180

##### Notes

Recorded in Burgio et al. (2000).

#### Doros profuges

(Harris, 1780)

647C23D8-73B1-5354-B5BA-BA028AB3CAE4

##### Materials

- **Type status:** Other material. **Occurrence:** sex: 1 female; occurrenceID: 64177DDF-927E-5283-83D9-1E02BE3E2399; **Taxon:** scientificName: Dorosprofuges (Harris, 1780); order: Diptera; family: Syrphidae; **Location:** country: Italy; locality: PNFC Foresta Lama Bertesca; decimalLatitude: 43.8314943; decimalLongitude: 11.8735824; geodeticDatum: WGS84; **Event:** samplingProtocol: Malaise Trap; verbatimEventDate: 30.vi.2004; **Record Level:** collectionID: AMSUB005142

##### Notes

Recorded in Burgio et al. (2000).

#### Epistrophe eligans

(Harris, 1780)

FE2CFA6B-8675-5064-9DAB-9E6ED48DDC96

##### Materials

- **Type status:** Other material. **Occurrence:** sex: 2 males; occurrenceID: 4ED451B2-B347-5865-9664-321C8256CBCF; **Taxon:** scientificName: Epistropheeligans (Harris, 1780); order: Diptera; family: Syrphidae; **Location:** country: Italy; locality: PNFC Foresta Lama Stazione Prati; decimalLatitude: 43.8313707; decimalLongitude: 11.8380049; geodeticDatum: WGS84; **Event:** samplingProtocol: Malaise Trap; verbatimEventDate: 26.v-6.vi.2003; **Record Level:** collectionID: AMSUB005189, AMSUB005190

##### Notes

Recorded in Burgio et al. (2000).

#### Epistrophe flava

Doczkal and Schmid, 1994

A40455CF-8F37-5A42-A4B8-D7DE838AF1E1

##### Notes

Recorded in Burgio et al. (2000).

#### Epistrophe grossulariae

(Meigen, 1822)

ADFA5B51-C9C1-547A-897C-8C5BD3F84C12

##### Notes

Recorded in Burgio et al. (2000).

#### Epistrophe nitidicollis

(Meigen, 1822)

150E7196-FC0E-558E-948E-5902877A84F2

##### Notes

Recorded in Burgio et al. (2000).

#### Episyrphus balteatus

(De Geer, 1776)

25AC184E-94B1-5058-8B5E-9575DD8FF072

##### Materials

- **Type status:** Other material. **Occurrence:** sex: 1 male, 1 female; occurrenceID: 5AF576B8-B5F3-5E25-AF56-8BB354E36C62; **Taxon:** scientificName: Episyrphusbalteatus (De Geer, 1776); order: Diptera; family: Syrphidae; **Location:** country: Italy; locality: PNFC Foresta Lama Fonte Cavalla; decimalLatitude: 43.8238539; decimalLongitude: 11.8835054; geodeticDatum: WGS84; **Event:** samplingProtocol: Malaise Trap; verbatimEventDate: 18.v-8.vi.2004; **Record Level:** collectionID: AMSUB005009, AMSUB005052
- **Type status:** Other material. **Occurrence:** sex: 1 female; occurrenceID: F2A5A251-77C0-5E0B-9F7A-C8D936451633; **Taxon:** scientificName: Episyrphusbalteatus (De Geer, 1776); order: Diptera; family: Syrphidae; **Location:** country: Italy; locality: PNFC Foresta Lama P. Saporita; decimalLatitude: 43.821774; decimalLongitude: 11.8802281; geodeticDatum: WGS84; **Event:** samplingProtocol: Malaise Trap; verbatimEventDate: 9.vii.2004; **Record Level:** collectionID: AMSUB005080
- **Type status:** Other material. **Occurrence:** sex: 2 males, 5 females; occurrenceID: 1BCBFA71-536D-5306-8E54-5F45850FC172; **Taxon:** scientificName: Episyrphusbalteatus (De Geer, 1776); order: Diptera; family: Syrphidae; **Location:** country: Italy; locality: PNFC Foresta Lama P. Saporita; decimalLatitude: 43.821774; decimalLongitude: 11.8802281; geodeticDatum: WGS84; **Event:** samplingProtocol: Malaise Trap; verbatimEventDate: 27.vii.2004; **Record Level:** collectionID: AMSUB005093, AMSUB005094, AMSUB005095, AMSUB005096, AMSUB005097, AMSUB005098, AMSUB005099
- **Type status:** Other material. **Occurrence:** sex: 1 male; occurrenceID: D6B7C3CC-6B04-5B66-A3AD-C588F85C746B; **Taxon:** scientificName: Episyrphusbalteatus (De Geer, 1776); order: Diptera; family: Syrphidae; **Location:** country: Italy; locality: PNFC Foresta Lama Bertesca; decimalLatitude: 43.8314943; decimalLongitude: 11.8735824; geodeticDatum: WGS84; **Event:** samplingProtocol: Malaise Trap; verbatimEventDate: 30.vi.2004; **Record Level:** collectionID: AMSUB005138
- **Type status:** Other material. **Occurrence:** sex: 1 male; occurrenceID: BBCC08A6-1762-516A-B269-D6DE6E234710; **Taxon:** scientificName: Episyrphusbalteatus (De Geer, 1776); order: Diptera; family: Syrphidae; **Location:** country: Italy; locality: PNFC Foresta Lama Bertesca; decimalLatitude: 43.8314943; decimalLongitude: 11.8735824; geodeticDatum: WGS84; **Event:** samplingProtocol: Malaise Trap; verbatimEventDate: 27.vii.2004; **Record Level:** collectionID: AMSUB005161
- **Type status:** Other material. **Occurrence:** sex: 3 males, 8 females; occurrenceID: 66267BBB-E410-5E19-A95E-AF5612487F66; **Taxon:** scientificName: Episyrphusbalteatus (De Geer, 1776); order: Diptera; family: Syrphidae; **Location:** country: Italy; locality: PNFC Foresta Lama Stazione Prati; decimalLatitude: 43.8313707; decimalLongitude: 11.8380049; geodeticDatum: WGS84; **Event:** samplingProtocol: Malaise Trap; verbatimEventDate: 6-16.viii.2003; **Record Level:** collectionID: AMSUB005293, AMSUB005294, AMSUB005295, AMSUB005296, AMSUB005297, AMSUB005298, AMSUB005299, AMSUB005300, AMSUB005301, AMSUB005302, AMSUB005303
- **Type status:** Other material. **Occurrence:** sex: 3 females; occurrenceID: A1BBD33D-48EC-5051-8F6B-99089588C2FB; **Taxon:** scientificName: Episyrphusbalteatus (De Geer, 1776); order: Diptera; family: Syrphidae; **Location:** country: Italy; locality: PNFC Foresta Lama Vetreria; decimalLatitude: 43.829961; decimalLongitude: 11.8380049; geodeticDatum: WGS84; **Event:** samplingProtocol: Malaise Trap; verbatimEventDate: 6-16.viii.2003; **Record Level:** collectionID: AMSUB005320, AMSUB005321, AMSUB005322
- **Type status:** Other material. **Occurrence:** sex: 1 female; occurrenceID: 31DE3AAB-7EC0-5F72-B8C3-CB6AA0CE302E; **Taxon:** scientificName: Episyrphusbalteatus (De Geer, 1776); order: Diptera; family: Syrphidae; **Location:** country: Italy; locality: PNFC Foresta Lama Vetreria; decimalLatitude: 43.829961; decimalLongitude: 11.8380049; geodeticDatum: WGS84; **Event:** samplingProtocol: Malaise Trap; verbatimEventDate: 3-14.vii.2003; **Record Level:** collectionID: AMSUB005325
- **Type status:** Other material. **Occurrence:** sex: 1 female, 6 exx; occurrenceID: 925401BB-FFEF-53C2-BE74-7A6BAB49B013; **Taxon:** scientificName: Episyrphusbalteatus (De Geer, 1776); order: Diptera; family: Syrphidae; **Location:** country: Italy; locality: PNFC Bucine (FC); decimalLatitude: 43.9594; decimalLongitude: 11.7097; geodeticDatum: WGS84; **Event:** samplingProtocol: Observation; verbatimEventDate: 12.viii.2023
- **Type status:** Other material. **Occurrence:** sex: 1 ex; occurrenceID: 77D77A38-8680-57E9-872A-8D3D91C96222; **Taxon:** scientificName: Episyrphusbalteatus (De Geer, 1776); order: Diptera; family: Syrphidae; **Location:** country: Italy; locality: PNFC Monte Falco (FI); decimalLatitude: 43.8773; decimalLongitude: 11.7107; geodeticDatum: WGS84; **Event:** samplingProtocol: Observation; verbatimEventDate: 17.ix.2023

##### Notes

Recorded in Burgio et al. (2000).

#### Eriozona syrphoides

(Fallén, 1817)

ADFC3675-D131-5B82-8A61-3C8DD008DB70

##### Materials

- **Type status:** Other material. **Occurrence:** sex: 1 female; occurrenceID: FFA99246-53EE-5060-B912-8920FE0B34D0; **Taxon:** scientificName: Eriozonasyrphoides (Fallén, 1817); order: Diptera; family: Syrphidae; **Location:** country: Italy; locality: PNFC Foresta Lama Bertesca; decimalLatitude: 43.8314943; decimalLongitude: 11.8735824; geodeticDatum: WGS84; **Event:** samplingProtocol: Malaise Trap; verbatimEventDate: 27.vii.2004; **Record Level:** collectionID: AMSUB005154

#### Eristalinus taeniops

(Wiedemann, 1818)

9E150A17-0603-567C-A405-533D3DD9250D

##### Materials

- **Type status:** Other material. **Occurrence:** occurrenceID: 667040A9-034A-54F5-AC6D-6826E82B6404; **Taxon:** scientificName: Eristalinustaeniops (Wiedemann, 1818); order: Diptera; family: Syrphidae; **Location:** country: Italy; locality: PNFC Bucine (FC); decimalLatitude: 43.9594; decimalLongitude: 11.7097; geodeticDatum: WGS84; **Event:** samplingProtocol: Observation; verbatimEventDate: 12.viii.2023

#### Eristalis arbustorum

(Linnaeus, 1758)

F9CF224E-A340-5203-B709-6EDF88618BF1

##### Notes

Recorded in Burgio et al. (2000).

#### Eristalis pertinax

(Scopoli, 1763)

C3C8114E-F865-5307-B290-16BB81742F90

##### Materials

- **Type status:** Other material. **Occurrence:** sex: 2 males; occurrenceID: 779C5D77-2373-5277-9E13-4B705B24ABB3; **Taxon:** scientificName: Eristalispertinax (Scopoli, 1763); order: Diptera; family: Syrphidae; **Location:** country: Italy; locality: PNFC Foresta Lama Stazione Prati; decimalLatitude: 43.8313707; decimalLongitude: 11.8380049; geodeticDatum: WGS84; **Event:** samplingProtocol: Malaise Trap; verbatimEventDate: 3-14.vii.2003; **Record Level:** collectionID: AMSUB005269, AMSUB005270
- **Type status:** Other material. **Occurrence:** sex: 1 female; occurrenceID: 3E1595E0-65F0-51B7-96FF-FC6D6E11AA0A; **Taxon:** scientificName: Eristalispertinax (Scopoli, 1763); order: Diptera; family: Syrphidae; **Location:** country: Italy; locality: PNFCFangacci (FC) 1400; decimalLatitude: 43.8103; decimalLongitude: 11.8489; geodeticDatum: WGS84; **Event:** samplingProtocol: Entomological Net; verbatimEventDate: 23.vii.1994; **Record Level:** collectionID: AMSUB005349
- **Type status:** Other material. **Occurrence:** sex: 1 male; occurrenceID: A2F67C94-5456-5E6A-ABFB-7E11858548AE; **Taxon:** scientificName: Eristalispertinax (Scopoli, 1763); order: Diptera; family: Syrphidae; **Location:** country: Italy; locality: PNFC Bucine (FC) 907m; decimalLatitude: 43.9594; decimalLongitude: 11.7097; geodeticDatum: WGS84; **Event:** samplingProtocol: Entomological Net; verbatimEventDate: 12.viii.2023; **Record Level:** collectionID: PNFCC000010

##### Notes

Recorded in Burgio et al. (2000).

#### Eristalis similis

(Fallén, 1817)

FCFEA7B4-87EF-5132-82BB-07E783F82EBE

##### Materials

- **Type status:** Other material. **Occurrence:** sex: 1 male; occurrenceID: 7EFB641A-B7BA-5107-961E-5CF92F0900CD; **Taxon:** scientificName: Eristalissimilis (Fallén, 1817); order: Diptera; family: Syrphidae; **Location:** country: Italy; locality: PNFC Foresta Lama Stazione Prati; decimalLatitude: 43.8313707; decimalLongitude: 11.8380049; geodeticDatum: WGS84; **Event:** samplingProtocol: Malaise Trap; verbatimEventDate: 3-14.vii.2003; **Record Level:** collectionID: AMSUB005271
- **Type status:** Other material. **Occurrence:** sex: 1 female; occurrenceID: F48459F5-AFD8-55B2-BA54-1444DCDF2275; **Taxon:** scientificName: Eristalissimilis (Fallén, 1817); order: Diptera; family: Syrphidae; **Location:** country: Italy; locality: PNFC Bucine (FC) 907m; decimalLatitude: 43.9594; decimalLongitude: 11.7097; geodeticDatum: WGS84; **Event:** samplingProtocol: Entomological Net; verbatimEventDate: 12.vi.2023; **Record Level:** collectionID: PNFCC000011
- **Type status:** Other material. **Occurrence:** sex: 1 male; occurrenceID: 729B3CCD-76DE-5634-9B1B-C998D1E05384; **Taxon:** scientificName: Eristalissimilis (Fallén, 1817); order: Diptera; family: Syrphidae; **Location:** country: Italy; locality: PNFC Monte Falco (FI) 1653m; decimalLatitude: 43.8773; decimalLongitude: 11.7107; geodeticDatum: WGS84; **Event:** samplingProtocol: Entomological Net; verbatimEventDate: 11.vii.2023; **Record Level:** collectionID: PNFCC000012
- **Type status:** Other material. **Occurrence:** sex: 2 males, 1 female; occurrenceID: 6CBEDD9E-CDF4-50F5-B720-EA7B7A511718; **Taxon:** scientificName: Eristalissimilis (Fallén, 1817); order: Diptera; family: Syrphidae; **Location:** country: Italy; locality: PNFC Monte Falco (FI) 1653m; decimalLatitude: 43.8773; decimalLongitude: 11.7107; geodeticDatum: WGS84; **Event:** samplingProtocol: Entomological Net; verbatimEventDate: 11.viii.2023; **Record Level:** collectionID: PNFCC000013, PNFCC000014, PNFCC000015
- **Type status:** Other material. **Occurrence:** sex: 1 male; occurrenceID: 64C6461C-734C-5D34-AF2F-44CB106C53D2; **Taxon:** scientificName: Eristalissimilis (Fallén, 1817); order: Diptera; family: Syrphidae; **Location:** country: Italy; locality: PNFC Bucine (FC) 907m; decimalLatitude: 43.9594; decimalLongitude: 11.7097; geodeticDatum: WGS84; **Event:** samplingProtocol: Entomological Net; verbatimEventDate: 12.viii.2023; **Record Level:** collectionID: PNFCC000016

#### Eristalis tenax

(Linnaeus, 1758)

8DC950E7-A6AC-549D-AA08-C838B5CDC443

##### Materials

- **Type status:** Other material. **Occurrence:** sex: 1 male, 1 female; occurrenceID: 2EC63ACF-7DF2-5265-A553-29AA5F06D7F5; **Taxon:** scientificName: Eristalistenax (Linnaeus, 1758); order: Diptera; family: Syrphidae; **Location:** country: Italy; locality: PNFC Fangacci (FC) 1400; decimalLatitude: 43.8103; decimalLongitude: 11.8489; geodeticDatum: WGS84; **Event:** samplingProtocol: Entomological Net; verbatimEventDate: 23.vii.1994; **Record Level:** collectionID: AMSUB005348, AMSUB005353
- **Type status:** Other material. **Occurrence:** sex: 2 males; occurrenceID: A78881EF-A89B-5E43-BFA8-B11B50CF10FE; **Taxon:** scientificName: Eristalistenax (Linnaeus, 1758); order: Diptera; family: Syrphidae; **Location:** country: Italy; locality: PNFCMonte Falco (FI) 1653m; decimalLatitude: 43.8773; decimalLongitude: 11.7107; geodeticDatum: WGS84; **Event:** samplingProtocol: Entomological Net; verbatimEventDate: 6.v.2023; **Record Level:** collectionID: PNFCC000017, PNFCC000018
- **Type status:** Other material. **Occurrence:** sex: 1 male; occurrenceID: 03922FEE-D727-505E-82F0-8FDE728D3D0A; **Taxon:** scientificName: Eristalistenax (Linnaeus, 1758); order: Diptera; family: Syrphidae; **Location:** country: Italy; locality: PNFC Bucine (FC) 907m; decimalLatitude: 43.9594; decimalLongitude: 11.7097; geodeticDatum: WGS84; **Event:** samplingProtocol: Entomological Net; verbatimEventDate: 12.vi.2023; **Record Level:** collectionID: PNFCC000019
- **Type status:** Other material. **Occurrence:** sex: 1 male; occurrenceID: 4897CF2E-A635-5965-8416-6E0D6962E8B9; **Taxon:** scientificName: Eristalistenax (Linnaeus, 1758); order: Diptera; family: Syrphidae; **Location:** country: Italy; locality: PNFC Monte Falco (FI) 1653m; decimalLatitude: 43.8773; decimalLongitude: 11.7107; geodeticDatum: WGS84; **Event:** samplingProtocol: Entomological Net; verbatimEventDate: 11.vii.2023; **Record Level:** collectionID: PNFCC000020
- **Type status:** Other material. **Occurrence:** sex: 1 male; occurrenceID: EE95C64E-847D-560D-AE03-8D33A1D2207E; **Taxon:** scientificName: Eristalistenax (Linnaeus, 1758); order: Diptera; family: Syrphidae; **Location:** country: Italy; locality: PNFC Bucine (FC) 907m; decimalLatitude: 43.9594; decimalLongitude: 11.7097; geodeticDatum: WGS84; **Event:** samplingProtocol: Entomological Net; verbatimEventDate: 12.vii.2023; **Record Level:** collectionID: PNFCC000021
- **Type status:** Other material. **Occurrence:** sex: 6 males, 1 female; occurrenceID: 3C9A70CD-497C-5E7A-84EC-66B40110F251; **Taxon:** scientificName: Eristalistenax (Linnaeus, 1758); order: Diptera; family: Syrphidae; **Location:** country: Italy; locality: PNFC Monte Falco (FI) 1653m; decimalLatitude: 43.8773; decimalLongitude: 11.7107; geodeticDatum: WGS84; **Event:** samplingProtocol: Entomological Net; verbatimEventDate: 11.viii.2023; **Record Level:** collectionID: PNFCC000022, PNFCC000023, PNFCC000024, PNFCC000025, PNFCC000026, PNFCC000027, PNFCC000028
- **Type status:** Other material. **Occurrence:** sex: 1 male, 1 female; occurrenceID: FD32A0CC-89DD-5330-BD61-4AAF822F241A; **Taxon:** scientificName: Eristalistenax (Linnaeus, 1758); order: Diptera; family: Syrphidae; **Location:** country: Italy; locality: PNFC Bucine (FC) 907m; decimalLatitude: 43.9594; decimalLongitude: 11.7097; geodeticDatum: WGS84; **Event:** samplingProtocol: Entomological Net; verbatimEventDate: 12.viii.2023; **Record Level:** collectionID: PNFCC000029, PNFCC000030

##### Notes

Recorded in Burgio et al. (2000).

#### Eumerus alpinus

Rondani, 1857

21EDB95F-3AEE-51A3-ACB3-A1131CC46EBF

##### Notes

Recorded in Burgio et al. (2000).

#### Eumerus strigatus

(Fallén, 1817)

3680092E-EAF5-5102-9A8C-4E8967B2F7FE

##### Notes

Recorded in Burgio et al. (2000).

#### Eupeodes bucculatus

(Rondani, 1857)

671DBEB7-6F12-521C-9DD2-D1D7D6551604

##### Materials

- **Type status:** Other material. **Occurrence:** sex: 1 female; occurrenceID: 2C576E6C-AADC-501B-912C-E491DDA059E5; **Taxon:** scientificName: Eupeodesbucculatus (Rondani, 1857); order: Diptera; family: Syrphidae; **Location:** country: Italy; locality: PNFC Bucine (FC) 907m; decimalLatitude: 43.9594; decimalLongitude: 11.7097; geodeticDatum: WGS84; **Event:** samplingProtocol: Entomological Net; verbatimEventDate: 12.viii.2023; **Record Level:** collectionID: PNFCC000031

#### Eupeodes corollae

(Fabricius, 1794)

71AB049F-C297-5DA5-8F2A-8E9FBD229A74

##### Materials

- **Type status:** Other material. **Occurrence:** sex: 1 male, 7 females; occurrenceID: 56B12EBC-8A5A-5347-B828-81350DE08440; **Taxon:** scientificName: Eupeodescorollae (Fabricius, 1794); order: Diptera; family: Syrphidae; **Location:** country: Italy; locality: PNFC Foresta Lama Fonte Cavalla; decimalLatitude: 43.8238539; decimalLongitude: 11.8835054; geodeticDatum: WGS84; **Event:** samplingProtocol: Malaise Trap; verbatimEventDate: 18.v-8.vi.2004; **Record Level:** collectionID: AMSUB005001, AMSUB005002, AMSUB005003, AMSUB005004, AMSUB005005, AMSUB005010, AMSUB005050, AMSUB005051
- **Type status:** Other material. **Occurrence:** sex: 1 male, 3 females; occurrenceID: 3DAB2F03-FA7D-58E7-B912-944874D217B1; **Taxon:** scientificName: Eupeodescorollae (Fabricius, 1794); order: Diptera; family: Syrphidae; **Location:** country: Italy; locality: PNFC Foresta Lama P. Saporita; decimalLatitude: 43.821774; decimalLongitude: 11.8802281; geodeticDatum: WGS84; **Event:** samplingProtocol: Malaise Trap; verbatimEventDate: 27.vii.2004; **Record Level:** collectionID: AMSUB005109, AMSUB005110, AMSUB005111, AMSUB005112
- **Type status:** Other material. **Occurrence:** sex: 2 males, 6 females; occurrenceID: EC507CFD-A14E-5B6F-BDC5-5EDE2FDF3C18; **Taxon:** scientificName: Eupeodescorollae (Fabricius, 1794); order: Diptera; family: Syrphidae; **Location:** country: Italy; locality: PNFC Foresta Lama Bertesca; decimalLatitude: 43.8314943; decimalLongitude: 11.8735824; geodeticDatum: WGS84; **Event:** samplingProtocol: Malaise Trap; verbatimEventDate: 30.vi.2004; **Record Level:** collectionID: AMSUB005130, AMSUB005131, AMSUB005132, AMSUB005133, AMSUB005134, AMSUB005135, AMSUB005136, AMSUB005137
- **Type status:** Other material. **Occurrence:** sex: 4 males; occurrenceID: E8C9E877-2DD8-5CFE-8BBD-0CEB093EF6BC; **Taxon:** scientificName: Eupeodescorollae (Fabricius, 1794); order: Diptera; family: Syrphidae; **Location:** country: Italy; locality: PNFC Foresta Lama Bertesca; decimalLatitude: 43.8314943; decimalLongitude: 11.8735824; geodeticDatum: WGS84; **Event:** samplingProtocol: Malaise Trap; verbatimEventDate: 27.vii.2004; **Record Level:** collectionID: AMSUB005155, AMSUB005156, AMSUB005157, AMSUB005158
- **Type status:** Other material. **Occurrence:** sex: 1 female; occurrenceID: C721F6CD-7339-554F-9715-FBF309A4A00E; **Taxon:** scientificName: Eupeodescorollae (Fabricius, 1794); order: Diptera; family: Syrphidae; **Location:** country: Italy; locality: PNFC Foresta Lama Stazione Prati; decimalLatitude: 43.8313707; decimalLongitude: 11.8380049; geodeticDatum: WGS84; **Event:** samplingProtocol: Malaise Trap; verbatimEventDate: 3-14.vii.2003; **Record Level:** collectionID: AMSUB005289
- **Type status:** Other material. **Occurrence:** sex: 1 female; occurrenceID: A24ECC8C-2CF2-5EDD-B324-1065DBB7A14D; **Taxon:** scientificName: Eupeodescorollae (Fabricius, 1794); order: Diptera; family: Syrphidae; **Location:** country: Italy; locality: PNFC Foresta Lama Stazione Prati; decimalLatitude: 43.8313707; decimalLongitude: 11.8380049; geodeticDatum: WGS84; **Event:** samplingProtocol: Malaise Trap; verbatimEventDate: 6-16.viii.2003; **Record Level:** collectionID: AMSUB005317
- **Type status:** Other material. **Occurrence:** sex: 1 male; occurrenceID: B353DE3A-3177-50D1-83BC-C1D9E137B97E; **Taxon:** scientificName: Eupeodescorollae (Fabricius, 1794); order: Diptera; family: Syrphidae; **Location:** country: Italy; locality: PNFC Piedimonte (FI) m. 500; decimalLatitude: 44.0924; decimalLongitude: 11.5185; geodeticDatum: WGS84; **Event:** samplingProtocol: Malaise Trap; verbatimEventDate: 15.vii.1994; **Record Level:** collectionID: AMSUB005340
- **Type status:** Other material. **Occurrence:** sex: 1 female; occurrenceID: 2B554A0D-FC40-5673-A7CE-40D922E4556C; **Taxon:** scientificName: Eupeodescorollae (Fabricius, 1794); order: Diptera; family: Syrphidae; **Location:** country: Italy; locality: PNFC Monte Falco (FI) 1653m; decimalLatitude: 43.8773; decimalLongitude: 11.7107; geodeticDatum: WGS84; **Event:** samplingProtocol: Malaise Trap; verbatimEventDate: 6.v.2023; **Record Level:** collectionID: PNFCC000032

#### Eupeodes latifasciatus

(Macquart, 1829)

B5DFEF37-8CB9-5144-ABC5-7175E011F906

##### Materials

- **Type status:** Other material. **Occurrence:** sex: 2 females; occurrenceID: 1E6DA93E-0457-5064-AA1F-25AFDF674A62; **Taxon:** scientificName: Eupeodeslatifasciatus (Macquart, 1829); order: Diptera; family: Syrphidae; **Location:** country: Italy; locality: PNFC Foresta Lama Fonte Cavalla; decimalLatitude: 43.8238539; decimalLongitude: 11.8835054; geodeticDatum: WGS84; **Event:** samplingProtocol: Malaise Trap; verbatimEventDate: 18.v-8.vi.2004; **Record Level:** collectionID: AMSUB005011, AMSUB005012
- **Type status:** Other material. **Occurrence:** sex: 1 female; occurrenceID: EC9FB3CF-2208-5447-A456-5DFE13C9DE29; **Taxon:** scientificName: Eupeodeslatifasciatus (Macquart, 1829); order: Diptera; family: Syrphidae; **Location:** country: Italy; locality: PNFC Foresta Lama P. Saporita; decimalLatitude: 43.821774; decimalLongitude: 11.8802281; geodeticDatum: WGS84; **Event:** samplingProtocol: Malaise Trap; verbatimEventDate: 9.vii.2004; **Record Level:** collectionID: AMSUB005083
- **Type status:** Other material. **Occurrence:** sex: 1 female; occurrenceID: 2B322A50-2F3C-53CF-9E30-E8145A172BAB; **Taxon:** scientificName: Eupeodeslatifasciatus (Macquart, 1829); order: Diptera; family: Syrphidae; **Location:** country: Italy; locality: PNFC Foresta Lama Bertesca; decimalLatitude: 43.8314943; decimalLongitude: 11.8735824; geodeticDatum: WGS84; **Event:** samplingProtocol: Malaise Trap; verbatimEventDate: 27.vii.2004; **Record Level:** collectionID: AMSUB005168
- **Type status:** Other material. **Occurrence:** sex: 2 females; occurrenceID: E4A0CD09-AD89-51C7-895E-2F8A42603667; **Taxon:** scientificName: Eupeodeslatifasciatus (Macquart, 1829); order: Diptera; family: Syrphidae; **Location:** country: Italy; locality: PNFC Foresta Lama Stazione Prati; decimalLatitude: 43.8313707; decimalLongitude: 11.8380049; geodeticDatum: WGS84; **Event:** samplingProtocol: Malaise Trap; verbatimEventDate: 3-14.vii.2003; **Record Level:** collectionID: AMSUB005290, AMSUB005291

##### Notes

Recorded in Burgio et al. (2000).

#### Eupeodes lucasi

(Marcos-Garcia & Laska, 1983)

487629E2-93F7-5F1A-A6D0-9CB1B4C5170D

##### Notes

Recorded in Burgio et al. (2000).

#### Eupeodes luniger

(Meigen, 1822)

770E714A-F5D1-5264-8AA9-D03E43935AFB

##### Materials

- **Type status:** Other material. **Occurrence:** sex: 1 male; occurrenceID: AA79845C-28D4-5FD9-9939-588129FC66F1; **Taxon:** scientificName: Eupeodesluniger (Meigen, 1822); order: Diptera; family: Syrphidae; **Location:** country: Italy; locality: PNFC Foresta Lama Fonte Cavalla; decimalLatitude: 43.8238539; decimalLongitude: 11.8835054; geodeticDatum: WGS84; **Event:** samplingProtocol: Malaise Trap; verbatimEventDate: 18.v-8.vi.2004; **Record Level:** collectionID: AMSUB005049
- **Type status:** Other material. **Occurrence:** sex: 1 male; occurrenceID: 1A051642-1CB7-5183-A5A3-593668255882; **Taxon:** scientificName: Eupeodesluniger (Meigen, 1822); order: Diptera; family: Syrphidae; **Location:** country: Italy; locality: PNFC Foresta Lama P. Saporita; decimalLatitude: 43.821774; decimalLongitude: 11.8802281; geodeticDatum: WGS84; **Event:** samplingProtocol: Malaise Trap; verbatimEventDate: 9.vii.2004; **Record Level:** collectionID: AMSUB005081
- **Type status:** Other material. **Occurrence:** sex: 6 males, 3 females; occurrenceID: 73C8B2E6-9128-562A-B0E9-C9589D9B6B0A; **Taxon:** scientificName: Eupeodesluniger (Meigen, 1822); order: Diptera; family: Syrphidae; **Location:** country: Italy; locality: PNFC Foresta Lama P. Saporita; decimalLatitude: 43.821774; decimalLongitude: 11.8802281; geodeticDatum: WGS84; **Event:** samplingProtocol: Malaise Trap; verbatimEventDate: 27.vii.2004; **Record Level:** collectionID: AMSUB005113, AMSUB005114, AMSUB005115, AMSUB005116, AMSUB005117, AMSUB005117, AMSUB005118, AMSUB005119, AMSUB005120, AMSUB005121
- **Type status:** Other material. **Occurrence:** sex: 5 females; occurrenceID: 3362D05A-EDE1-5F5C-9FBF-D04C6C1EB855; **Taxon:** scientificName: Eupeodesluniger (Meigen, 1822); order: Diptera; family: Syrphidae; **Location:** country: Italy; locality: PNFC Foresta Lama Bertesca; decimalLatitude: 43.8314943; decimalLongitude: 11.8735824; geodeticDatum: WGS84; **Event:** samplingProtocol: Malaise Trap; verbatimEventDate: 27.vii.2004; **Record Level:** collectionID: AMSUB005167, AMSUB005169, AMSUB005170, AMSUB005171, AMSUB005172
- **Type status:** Other material. **Occurrence:** sex: 1 male, 2 females; occurrenceID: 5035FC79-26DA-5A07-97BD-3A910B55E01E; **Taxon:** scientificName: Eupeodesluniger (Meigen, 1822); order: Diptera; family: Syrphidae; **Location:** country: Italy; locality: PNFC Foresta Lama Stazione Prati; decimalLatitude: 43.8313707; decimalLongitude: 11.8380049; geodeticDatum: WGS84; **Event:** samplingProtocol: Malaise Trap; verbatimEventDate: 3-14.vii.2003; **Record Level:** collectionID: AMSUB005286, AMSUB005287, AMSUB005288
- **Type status:** Other material. **Occurrence:** sex: 2 females; occurrenceID: 3D7C7CC8-5307-5A8A-8CE9-F4C8956B5F90; **Taxon:** scientificName: Eupeodesluniger (Meigen, 1822); order: Diptera; family: Syrphidae; **Location:** country: Italy; locality: PNFC Foresta Lama Stazione Prati; decimalLatitude: 43.8313707; decimalLongitude: 11.8380049; geodeticDatum: WGS84; **Event:** samplingProtocol: Malaise Trap; verbatimEventDate: 6-16.viii.2003; **Record Level:** collectionID: AMSUB005315, AMSUB005316
- **Type status:** Other material. **Occurrence:** sex: 1 female; occurrenceID: 340F7F2C-3D2D-5841-9336-2C219B810948; **Taxon:** scientificName: Eupeodesluniger (Meigen, 1822); order: Diptera; family: Syrphidae; **Location:** country: Italy; locality: PNFC Le Cullacce Campigna m. 1041; decimalLatitude: 43.8628; decimalLongitude: 11.7527; geodeticDatum: WGS84; **Event:** samplingProtocol: Entomological Net; verbatimEventDate: 18.vi.1994; **Record Level:** collectionID: AMSUB005344
- **Type status:** Other material. **Occurrence:** sex: 1 female; occurrenceID: 1617F1CF-CABD-5A22-B31D-42ECF17E6DA5; **Taxon:** scientificName: Eupeodesluniger (Meigen, 1822); order: Diptera; family: Syrphidae; **Location:** country: Italy; locality: PNFC Monte Falco (FI) 1653m; decimalLatitude: 43.8773; decimalLongitude: 11.7107; geodeticDatum: WGS84; **Event:** samplingProtocol: Entomological Net; verbatimEventDate: 11.vi.2023; **Record Level:** collectionID: PNFCC000033
- **Type status:** Other material. **Occurrence:** sex: 1 male; occurrenceID: 2EC9EBDB-D252-5603-840E-E75ECE19E3B1; **Taxon:** scientificName: Eupeodesluniger (Meigen, 1822); order: Diptera; family: Syrphidae; **Location:** country: Italy; locality: PNFC Monte Falco (FI) 1653m; decimalLatitude: 43.8773; decimalLongitude: 11.7107; geodeticDatum: WGS84; **Event:** samplingProtocol: Entomological Net; verbatimEventDate: 11.viii.2023; **Record Level:** collectionID: PNFCC000034
- **Type status:** Other material. **Occurrence:** sex: 1 female; occurrenceID: 2E8E78C3-DAA4-58DB-8002-54FE5E2C67B4; **Taxon:** scientificName: Eupeodesluniger (Meigen, 1822); order: Diptera; family: Syrphidae; **Location:** country: Italy; locality: PNFC Bucine (FC) 907m; decimalLatitude: 43.9594; decimalLongitude: 11.7097; geodeticDatum: WGS84; **Event:** samplingProtocol: Entomological Net; verbatimEventDate: 12.viii.2023; **Record Level:** collectionID: PNFCC000035

##### Notes

Recorded in Burgio et al. (2000).

#### Eupeodes nitens

(Zetterstedt, 1843)

2EAE0B72-F508-5BA9-B0BA-8EAFC9AF39FB

##### Notes

Recorded in Burgio et al. (2000).

#### Fagisyrphus cinctus

(Fallén, 1817)

B7476FEE-1C3F-581C-9BD2-7D444131BC80

##### Materials

- **Type status:** Other material. **Occurrence:** sex: 1 female; occurrenceID: BE1BC9EF-9E23-53A2-A8BD-5371E672B3AB; **Taxon:** scientificName: Fagisyrphuscinctus (Fallén, 1817); order: Diptera; family: Syrphidae; **Location:** country: Italy; locality: PNFC Foresta Lama Fonte Cavalla; decimalLatitude: 43.8238539; decimalLongitude: 11.8835054; geodeticDatum: WGS84; **Event:** samplingProtocol: Malaise Trap; verbatimEventDate: 18.v-8.vi.2004; **Record Level:** collectionID: AMSUB005013
- **Type status:** Other material. **Occurrence:** sex: 1 male; occurrenceID: F8BF128F-86A7-5356-BB09-9D2AE684ACCE; **Taxon:** scientificName: Fagisyrphuscinctus (Fallén, 1817); order: Diptera; family: Syrphidae; **Location:** country: Italy; locality: PNFC Foresta Lama P. Saporita; decimalLatitude: 43.821774; decimalLongitude: 11.8802281; geodeticDatum: WGS84; **Event:** samplingProtocol: Malaise Trap; verbatimEventDate: 27.vii.2004; **Record Level:** collectionID: AMSUB005125

##### Notes

Recorded in Burgio et al. (2000).

#### Ferdinandea cuprea

(Scopoli, 1763)

89927A70-4106-5AB4-ADD5-3F833B77EECF

##### Notes

Recorded in Burgio et al. (2000).

#### Heringia heringi

(Zetterstedt, 1843)

6630FAEE-EFC3-5467-9E0C-967B35FEC656

##### Materials

- **Type status:** Other material. **Occurrence:** sex: 1 male; occurrenceID: 8B05B333-E72B-5FCF-80D9-352582510D43; **Taxon:** scientificName: Heringiaheringi (Zetterstedt, 1843); order: Diptera; family: Syrphidae; **Location:** country: Italy; locality: PNFC Foresta Lama Stazione Prati; decimalLatitude: 43.8313707; decimalLongitude: 11.8380049; geodeticDatum: WGS84; **Event:** samplingProtocol: Malaise Trap; verbatimEventDate: 6-16.viii.2003; **Record Level:** collectionID: AMSUB005319

#### Lapposyrphus lapponicus

(Zetterstedt, 1838)

D554B5B2-F072-509B-AF69-4DE2623F1E85

##### Materials

- **Type status:** Other material. **Occurrence:** sex: 1 female; occurrenceID: 69AFAE7D-CBEB-5FFC-9C03-64C1A4B3B90A; **Taxon:** scientificName: Lapposyrphuslapponicus (Zetterstedt, 1838); order: Diptera; family: Syrphidae; **Location:** country: Italy; locality: PNFC Foresta Lama Grigiole 800 m; decimalLatitude: 43.832961; decimalLongitude: 11.8704871; geodeticDatum: WGS84; **Event:** samplingProtocol: Malaise Trap; verbatimEventDate: 6.viii.2003; **Record Level:** collectionID: AMSUB005182

##### Notes

Recorded in Burgio et al. (2000).

#### Leucozona lucorum

(Linnaeus, 1758)

4D8C7A77-6141-507A-A213-5802E78D9C67

##### Notes

Recorded in Burgio et al. (2000).

#### Matsumyia berberina

(Fabricius, 1805)

7133F810-BEB3-5CD6-9A5B-7BC66DAA02F6

##### Notes

Recorded in Burgio et al. (2000).

#### Megasyrphus erraticus

(Linnaeus, 1758)

96400E22-EE29-5C68-AB93-8555048254D7

##### Notes

Recorded in Burgio et al. (2000).

#### Melangyna compositarum

(Verrall, 1873)

5FBB1589-9C8F-5853-A4DF-D18FFB301C49

##### Materials

- **Type status:** Other material. **Occurrence:** sex: 1 female; occurrenceID: CC365491-2654-5562-AB05-AB94253444E7; **Taxon:** scientificName: Melangynacompositarum (Verrall, 1873); order: Diptera; family: Syrphidae; **Location:** country: Italy; locality: PNFC Monte Falco (FI) 1653m; decimalLatitude: 43.8773; decimalLongitude: 11.7107; geodeticDatum: WGS84; **Event:** samplingProtocol: Entomological Net; verbatimEventDate: 11.vi.2023; **Record Level:** collectionID: PNFCC000036

#### Melangyna umbellatarum

(Fabricius, 1794)

B1F979C9-A3D9-58EB-BA9B-DD8A4D36A753

##### Notes

Recorded in Burgio et al. (2000).

#### Melanogaster nuda

(Macquart, 1829)

57BE5D39-E96C-5E3F-8B2C-564273499BB1

##### Materials

- **Type status:** Other material. **Occurrence:** sex: 2 males; occurrenceID: 44439768-2015-5EB1-9A1A-E34368E54335; **Taxon:** scientificName: Melanogasternuda (Macquart, 1829); order: Diptera; family: Syrphidae; **Location:** country: Italy; locality: PNFC Bucine (FC) 907m; decimalLatitude: 43.9594; decimalLongitude: 11.7097; geodeticDatum: WGS84; **Event:** samplingProtocol: Entomological Net; verbatimEventDate: 7.v.2023; **Record Level:** collectionID: PNFCC000037, PNFCC000038

#### Melanostoma mellinum

(Linnaeus, 1758)

FB386D72-40CB-5F07-A063-8E69DCD4760E

##### Materials

- **Type status:** Other material. **Occurrence:** sex: 1 male; occurrenceID: 50024436-D43D-574E-84DC-7986F119786E; **Taxon:** scientificName: Melanostomamellinum (Linnaeus, 1758); order: Diptera; family: Syrphidae; **Location:** country: Italy; locality: PNFC Foresta Lama Fonte Cavalla; decimalLatitude: 43.8238539; decimalLongitude: 11.8835054; geodeticDatum: WGS84; **Event:** samplingProtocol: Malaise Trap; verbatimEventDate: 18.v-8.vi.2004; **Record Level:** collectionID: AMSUB005055
- **Type status:** Other material. **Occurrence:** sex: 1 male; occurrenceID: CDF21E51-1C14-5ABF-931C-DCC0928BD52B; **Taxon:** scientificName: Melanostomamellinum (Linnaeus, 1758); order: Diptera; family: Syrphidae; **Location:** country: Italy; locality: PNFC Foresta Lama Bertesca; decimalLatitude: 43.8314943; decimalLongitude: 11.8735824; geodeticDatum: WGS84; **Event:** samplingProtocol: Malaise Trap; verbatimEventDate: 30.vi.2004; **Record Level:** collectionID: AMSUB005128
- **Type status:** Other material. **Occurrence:** sex: 1 female; occurrenceID: 00ABC36C-2A76-5EBE-A103-F99ABAC97E71; **Taxon:** scientificName: Melanostomamellinum (Linnaeus, 1758); order: Diptera; family: Syrphidae; **Location:** country: Italy; locality: PNFC Foresta Lama Stazione Prati; decimalLatitude: 43.8313707; decimalLongitude: 11.8380049; geodeticDatum: WGS84; **Event:** samplingProtocol: Malaise Trap; verbatimEventDate: 26.v-6.vi.2003; **Record Level:** collectionID: AMSUB005211
- **Type status:** Other material. **Occurrence:** sex: 1 female; occurrenceID: 35ADBC4A-B330-570A-A481-BEE9CCB49777; **Taxon:** scientificName: Melanostomamellinum (Linnaeus, 1758); order: Diptera; family: Syrphidae; **Location:** country: Italy; locality: PNFC Bucine (FC) 907m; decimalLatitude: 43.9594; decimalLongitude: 11.7097; geodeticDatum: WGS84; **Event:** samplingProtocol: Entomological Net; verbatimEventDate: 12.vi.2023; **Record Level:** collectionID: PNFCC000039
- **Type status:** Other material. **Occurrence:** sex: 1 female; occurrenceID: A328414D-1C14-570E-9992-E7B1CC69D0FF; **Taxon:** scientificName: Melanostomamellinum (Linnaeus, 1758); order: Diptera; family: Syrphidae; **Location:** country: Italy; locality: PNFC Bucine (FC) 907m; decimalLatitude: 43.9594; decimalLongitude: 11.7097; geodeticDatum: WGS84; **Event:** samplingProtocol: Entomological Net; verbatimEventDate: 12.vii.2023; **Record Level:** collectionID: PNFCC000040
- **Type status:** Other material. **Occurrence:** sex: 1 male; occurrenceID: A65F3A77-907B-59B3-B95F-3A7156708E69; **Taxon:** scientificName: Melanostomamellinum (Linnaeus, 1758); order: Diptera; family: Syrphidae; **Location:** country: Italy; locality: PNFC Bucine (FC) 907m; decimalLatitude: 43.9594; decimalLongitude: 11.7097; geodeticDatum: WGS84; **Event:** samplingProtocol: Entomological Net; verbatimEventDate: 12.vii.2023; **Record Level:** collectionID: PNFCC000041

##### Notes

Recorded in Burgio et al. (2000).

#### Melanostoma scalare

(Linnaeus, 1794)

AE0C5D11-1AEB-5DC4-A2B6-66E9FEA2E9D7

##### Materials

- **Type status:** Other material. **Occurrence:** sex: 1 female; occurrenceID: 33AA6573-1EE8-553D-AFFE-3A95C5E09C95; **Taxon:** scientificName: Melanostomascalare (Linnaeus, 1794); order: Diptera; family: Syrphidae; **Location:** country: Italy; locality: PNFC Foresta Lama P. Saporita; decimalLatitude: 43.821774; decimalLongitude: 11.8802281; geodeticDatum: WGS84; **Event:** samplingProtocol: Malaise Trap; verbatimEventDate: 9.vii.2004; **Record Level:** collectionID: AMSUB005084
- **Type status:** Other material. **Occurrence:** sex: 1 male; occurrenceID: 4138679B-C9D3-5185-A4FB-54B44FAEBB5C; **Taxon:** scientificName: Melanostomascalare (Linnaeus, 1794); order: Diptera; family: Syrphidae; **Location:** country: Italy; locality: PNFC Foresta Lama Stazione Prati; decimalLatitude: 43.8313707; decimalLongitude: 11.8380049; geodeticDatum: WGS84; **Event:** samplingProtocol: Malaise Trap; verbatimEventDate: 3-14.vii.2003; **Record Level:** collectionID: AMSUB005281
- **Type status:** Other material. **Occurrence:** sex: 1 female; occurrenceID: BEB1C05C-7CEF-5A9E-9969-160E4DA49988; **Taxon:** scientificName: Melanostomascalare (Linnaeus, 1794); order: Diptera; family: Syrphidae; **Location:** country: Italy; locality: PNFC Monte Falco (FI) 1653m; decimalLatitude: 43.8773; decimalLongitude: 11.7107; geodeticDatum: WGS84; **Event:** samplingProtocol: Entomological Net; verbatimEventDate: 17.ix.2023; **Record Level:** collectionID: PNFCC000042

##### Notes

Recorded in Burgio et al. (2000).

#### Meligramma cingulata

(Egger, 1860)

FF94AD2A-3B5F-59F3-8EB8-C8A15B4FEF6E

##### Materials

- **Type status:** Other material. **Occurrence:** sex: 3 males; occurrenceID: FBA388CD-08AF-515A-BE28-1B04DAD99EAB; **Taxon:** scientificName: Meligrammacingulata (Egger, 1860); order: Diptera; family: Syrphidae; **Location:** country: Italy; locality: PNFC Foresta Lama Stazione Vetreria; decimalLatitude: 43.829961; decimalLongitude: 11.8418409; geodeticDatum: WGS84; **Event:** samplingProtocol: Malaise Trap; verbatimEventDate: 26.v-6.vi.2003; **Record Level:** collectionID: AMSUB005185, AMSUB005186, AMSUB005187
- **Type status:** Other material. **Occurrence:** sex: 14 males, 2 females; occurrenceID: 80AD7A49-39B9-5720-8537-F24A46EE7CDB; **Taxon:** scientificName: Meligrammacingulata (Egger, 1860); order: Diptera; family: Syrphidae; **Location:** country: Italy; locality: PNFC Foresta Lama Stazione Prati; decimalLatitude: 43.8313707; decimalLongitude: 11.8380049; geodeticDatum: WGS84; **Event:** samplingProtocol: Malaise Trap; verbatimEventDate: 26.v-6.vi.2003; **Record Level:** collectionID: AMSUB005195, AMSUB005196, AMSUB005197, AMSUB005198, AMSUB005199, AMSUB005200, AMSUB005201, AMSUB005202, AMSUB005203, AMSUB005204, AMSUB005205, AMSUB005206, AMSUB005207, AMSUB005208, AMSUB005209, AMSUB005212

##### Notes

Recorded in Burgio et al. (2000).

#### Meliscaeva auricollis

(Meigen, 1822)

DE8C6F6C-1CE4-5874-974B-7DBB330668A0

##### Materials

- **Type status:** Other material. **Occurrence:** sex: 3 males; occurrenceID: D4E5CB2B-B0F1-514B-A4CA-15219B722954; **Taxon:** scientificName: Meliscaevaauricollis (Meigen, 1822); order: Diptera; family: Syrphidae; **Location:** country: Italy; locality: PNFC Foresta Lama P. Saporita; decimalLatitude: 43.821774; decimalLongitude: 11.8802281; geodeticDatum: WGS84; **Event:** samplingProtocol: Malaise Trap; verbatimEventDate: 27.vii.2004; **Record Level:** collectionID: AMSUB005028, AMSUB005029, AMSUB005030
- **Type status:** Other material. **Occurrence:** sex: 3 males; occurrenceID: E924A424-5C6E-5856-9123-74376EEF18DD; **Taxon:** scientificName: Meliscaevaauricollis (Meigen, 1822); order: Diptera; family: Syrphidae; **Location:** country: Italy; locality: PNFC Foresta Lama Fonte Cavalla; decimalLatitude: 43.8238539; decimalLongitude: 11.8835054; geodeticDatum: WGS84; **Event:** samplingProtocol: Malaise Trap; verbatimEventDate: 18.v-8.vi.2004; **Record Level:** collectionID: AMSUB005031, AMSUB005032, AMSUB005033
- **Type status:** Other material. **Occurrence:** sex: 5 males; occurrenceID: 1AD721EC-9F1E-5A03-BC87-A22F4BA36F65; **Taxon:** scientificName: Meliscaevaauricollis (Meigen, 1822); order: Diptera; family: Syrphidae; **Location:** country: Italy; locality: PNFC Foresta Lama P. Saporita; decimalLatitude: 43.821774; decimalLongitude: 11.8802281; geodeticDatum: WGS84; **Event:** samplingProtocol: Malaise Trap; verbatimEventDate: 9.vii.2004; **Record Level:** collectionID: AMSUB005065, AMSUB005066, AMSUB005067, AMSUB005068, AMSUB005069
- **Type status:** Other material. **Occurrence:** sex: 3 males; occurrenceID: FB283696-BFB5-5CDE-9F68-A16F5D819DB7; **Taxon:** scientificName: Meliscaevaauricollis (Meigen, 1822); order: Diptera; family: Syrphidae; **Location:** country: Italy; locality: PNFC Foresta Lama P. Saporita; decimalLatitude: 43.821774; decimalLongitude: 11.8802281; geodeticDatum: WGS84; **Event:** samplingProtocol: Malaise Trap; verbatimEventDate: 27.vii.2004; **Record Level:** collectionID: AMSUB005101, AMSUB005102, AMSUB005103
- **Type status:** Other material. **Occurrence:** sex: 3 males; occurrenceID: AC587372-01E8-5488-87F3-2CC10CE13C60; **Taxon:** scientificName: Meliscaevaauricollis (Meigen, 1822); order: Diptera; family: Syrphidae; **Location:** country: Italy; locality: PNFC Foresta Lama Bertesca; decimalLatitude: 43.8314943; decimalLongitude: 11.8735824; geodeticDatum: WGS84; **Event:** samplingProtocol: Malaise Trap; verbatimEventDate: 27.vii.2004; **Record Level:** collectionID: AMSUB005164, AMSUB005165, AMSUB005166
- **Type status:** Other material. **Occurrence:** sex: 1 male; occurrenceID: 215AD1EC-1279-5CB0-A321-00ECAA4BB7F4; **Taxon:** scientificName: Meliscaevaauricollis (Meigen, 1822); order: Diptera; family: Syrphidae; **Location:** country: Italy; locality: PNFC Foresta Lama Stazione Prati; decimalLatitude: 43.8313707; decimalLongitude: 11.8380049; geodeticDatum: WGS84; **Event:** samplingProtocol: Malaise Trap; verbatimEventDate: 3-14.vii.2003; **Record Level:** collectionID: AMSUB005277
- **Type status:** Other material. **Occurrence:** sex: 1 female; occurrenceID: C50AF17F-36E9-548E-A915-5DBCFBDEF756; **Taxon:** scientificName: Meliscaevaauricollis (Meigen, 1822); order: Diptera; family: Syrphidae; **Location:** country: Italy; locality: PNFC Monte Falco (FI) 1653m; decimalLatitude: 43.8773; decimalLongitude: 11.7107; geodeticDatum: WGS84; **Event:** samplingProtocol: Malaise Trap; verbatimEventDate: 11.vi.2023; **Record Level:** collectionID: PNFCC000043
- **Type status:** Other material. **Occurrence:** sex: 3 males, 1 female; occurrenceID: DB09B89D-0695-5DCC-A16F-242C1274D9D6; **Taxon:** scientificName: Meliscaevaauricollis (Meigen, 1822); order: Diptera; family: Syrphidae; **Location:** country: Italy; locality: PNFC Bucine (FC) 907m; decimalLatitude: 43.9594; decimalLongitude: 11.7097; geodeticDatum: WGS84; **Event:** samplingProtocol: Malaise Trap; verbatimEventDate: 12.vi.2023; **Record Level:** collectionID: PNFCC000044, PNFCC000045, PNFCC000046, PNFCC000047

##### Notes

Recorded in Burgio et al. (2000).

#### Meliscaeva cinctella

(Zetterstedt, 1843)

EBA93DC6-7654-5C43-826A-984C300D0152

##### Materials

- **Type status:** Other material. **Occurrence:** sex: 12 males, 3 females; occurrenceID: 56B5D639-9E6F-55B9-BAD7-70F54A818015; **Taxon:** scientificName: Meliscaevacinctella (Zetterstedt, 1843); order: Diptera; family: Syrphidae; **Location:** country: Italy; locality: PNFC Foresta Lama Fonte Cavalla; decimalLatitude: 43.8238539; decimalLongitude: 11.8835054; geodeticDatum: WGS84; **Event:** samplingProtocol: Malaise Trap; verbatimEventDate: 18.v-8.vi.2004; **Record Level:** collectionID: AMSUB005034, AMSUB005035, AMSUB005036, AMSUB005037, AMSUB005038, AMSUB005039, AMSUB005040, AMSUB005041, AMSUB005042, AMSUB005043, AMSUB005044, AMSUB005045, AMSUB005046, AMSUB005047, AMSUB005048
- **Type status:** Other material. **Occurrence:** sex: 4 males, 5 females; occurrenceID: F4156413-EBDE-50E0-8BD1-63ADF20FA012; **Taxon:** scientificName: Meliscaevacinctella (Zetterstedt, 1843); order: Diptera; family: Syrphidae; **Location:** country: Italy; locality: PNFC Foresta Lama P. Saporita; decimalLatitude: 43.821774; decimalLongitude: 11.8802281; geodeticDatum: WGS84; **Event:** samplingProtocol: Malaise Trap; verbatimEventDate: 9.vii.2004; **Record Level:** collectionID: AMSUB005070, AMSUB005071, AMSUB005072, AMSUB005073, AMSUB005074, AMSUB005075, AMSUB005076, AMSUB005077, AMSUB005078
- **Type status:** Other material. **Occurrence:** sex: 4 males, 1 females; occurrenceID: 6E8C5E70-166C-568E-A875-40B10735251C; **Taxon:** scientificName: Meliscaevacinctella (Zetterstedt, 1843); order: Diptera; family: Syrphidae; **Location:** country: Italy; locality: PNFC Foresta Lama P. Saporita; decimalLatitude: 43.821774; decimalLongitude: 11.8802281; geodeticDatum: WGS84; **Event:** samplingProtocol: Malaise Trap; verbatimEventDate: 27.vii.2004; **Record Level:** collectionID: AMSUB005104, AMSUB005105, AMSUB005106, AMSUB005107, AMSUB005108
- **Type status:** Other material. **Occurrence:** sex: 1 male; occurrenceID: 4461B541-DCA5-5AB2-93DA-99884411A3BD; **Taxon:** scientificName: Meliscaevacinctella (Zetterstedt, 1843); order: Diptera; family: Syrphidae; **Location:** country: Italy; locality: PNFC Foresta Lama Stazione Vetreria; decimalLatitude: 43.829961; decimalLongitude: 11.8418409; geodeticDatum: WGS84; **Event:** samplingProtocol: Malaise Trap; verbatimEventDate: 26.v-6.vi.2003; **Record Level:** collectionID: AMSUB005184
- **Type status:** Other material. **Occurrence:** sex: 1 male, 1 female; occurrenceID: A7D9067A-30D2-5A5F-B75F-5F40C754BC4A; **Taxon:** scientificName: Meliscaevacinctella (Zetterstedt, 1843); order: Diptera; family: Syrphidae; **Location:** country: Italy; locality: PNFC Foresta Lama Stazione Prati; decimalLatitude: 43.8313707; decimalLongitude: 11.8380049; geodeticDatum: WGS84; **Event:** samplingProtocol: Malaise Trap; verbatimEventDate: 26.v-6.vi.2003; **Record Level:** collectionID: AMSUB005213, AMSUB005213
- **Type status:** Other material. **Occurrence:** sex: 1 male, 4 females; occurrenceID: A08CB63A-B465-58DF-BA1E-F4C85C8AE52D; **Taxon:** scientificName: Meliscaevacinctella (Zetterstedt, 1843); order: Diptera; family: Syrphidae; **Location:** country: Italy; locality: PNFC Foresta Lama Stazione Prati; decimalLatitude: 43.8313707; decimalLongitude: 11.8380049; geodeticDatum: WGS84; **Event:** samplingProtocol: Malaise Trap; verbatimEventDate: 3-14.vii.2003; **Record Level:** collectionID: AMSUB005272, AMSUB005273, AMSUB005274, AMSUB005275, AMSUB005277
- **Type status:** Other material. **Occurrence:** sex: 1 female; occurrenceID: 42E75EF7-76E6-5FA7-ACB2-1FE84B5748B2; **Taxon:** scientificName: Meliscaevacinctella (Zetterstedt, 1843); order: Diptera; family: Syrphidae; **Location:** country: Italy; locality: PNFC Foresta Lama Vetreria; decimalLatitude: 43.829961; decimalLongitude: 11.8380049; geodeticDatum: WGS84; **Event:** samplingProtocol: Malaise Trap; verbatimEventDate: 6-16.viii.2003; **Record Level:** collectionID: AMSUB005323
- **Type status:** Other material. **Occurrence:** sex: 1 female; occurrenceID: 9215D765-7186-59E2-8AC4-582D099A68B3; **Taxon:** scientificName: Meliscaevacinctella (Zetterstedt, 1843); order: Diptera; family: Syrphidae; **Location:** country: Italy; locality: PNFC Foresta Lama Vetreria; decimalLatitude: 43.829961; decimalLongitude: 11.8380049; geodeticDatum: WGS84; **Event:** samplingProtocol: Malaise Trap; verbatimEventDate: 3-14.vii.2003; **Record Level:** collectionID: AMSUB005324

##### Notes

Recorded in Burgio et al. (2000).

#### Merodon aberrans

Egger, 1860

132F0F5B-3F6C-5EDE-9506-038892CFC817

##### Notes

Recorded in Burgio et al. (2000).

#### Merodon aureus

Fabricius, 1805

12C29F4D-FE46-52C5-B935-3D678D73C820

##### Materials

- **Type status:** Other material. **Occurrence:** sex: 1 male; occurrenceID: D105BAE2-3F72-502D-9437-F33629AA467F; **Taxon:** scientificName: Merodonaureus Fabricius, 1805; order: Diptera; family: Syrphidae; **Location:** country: Italy; locality: PNFC Monte Falco (FI) 1653m; decimalLatitude: 43.8773; decimalLongitude: 11.7107; geodeticDatum: WGS84; **Event:** samplingProtocol: Entomological Net; verbatimEventDate: 11.vi.2023; **Record Level:** collectionID: PNFCC000048

##### Notes

Recorded in Burgio et al. (2000).

#### Microdon analis

(Macquart, 1842)

D6D7A6B8-2924-55F8-BFBB-ECDE056C4028

##### Materials

- **Type status:** Other material. **Occurrence:** sex: 2 females; occurrenceID: 1B23A56F-928C-539F-81AA-AAC2FA0A047C; **Taxon:** scientificName: Microdonanalis (Macquart, 1842); order: Diptera; family: Syrphidae; **Location:** country: Italy; locality: PNFC Foresta Lama Stazione Prati; decimalLatitude: 43.8313707; decimalLongitude: 11.8380049; geodeticDatum: WGS84; **Event:** samplingProtocol: Malaise Trap; verbatimEventDate: 26.v-6.vi.2003; **Record Level:** collectionID: AMSUB005191, AMSUB005192

#### Microdon mutabilis

(Linnaeus, 1758)

E4998D5F-63D2-5029-A7AB-CF1BBB9E92D7

##### Notes

Recorded in Burgio et al. (2000).

#### Myathropa florea

(Linnaeus, 1758)

975C85CB-8A0B-5FAB-8B60-14AE58BEA398

##### Materials

- **Type status:** Other material. **Occurrence:** sex: 1 female; occurrenceID: 29BCA54C-7C82-5A3C-A9EC-D2C319270CBA; **Taxon:** scientificName: Myathropaflorea (Linnaeus, 1758); order: Diptera; family: Syrphidae; **Location:** country: Italy; locality: PNFC Foresta Lama Fonte Cavalla; decimalLatitude: 43.8238539; decimalLongitude: 11.8835054; geodeticDatum: WGS84; **Event:** samplingProtocol: Malaise Trap; verbatimEventDate: 18.v-8.vi.2004; **Record Level:** collectionID: AMSUB005057
- **Type status:** Other material. **Occurrence:** sex: 2 females; occurrenceID: 85F05C32-72A2-559E-B885-75DB33389704; **Taxon:** scientificName: Myathropaflorea (Linnaeus, 1758); order: Diptera; family: Syrphidae; **Location:** country: Italy; locality: PNFC Fangacci (FC) 1400; decimalLatitude: 43.8103; decimalLongitude: 11.8489; geodeticDatum: WGS84; **Event:** samplingProtocol: Entomological Net; verbatimEventDate: 23.vii.1994; **Record Level:** collectionID: AMSUB005350, AMSUB005351
- **Type status:** Other material. **Occurrence:** sex: 1 female; occurrenceID: 9C28303C-1729-5DAE-86AA-33C9C3A87107; **Taxon:** scientificName: Myathropaflorea (Linnaeus, 1758); order: Diptera; family: Syrphidae; **Location:** country: Italy; locality: PNFC Bucine (FC) 907m; decimalLatitude: 43.9594; decimalLongitude: 11.7097; geodeticDatum: WGS84; **Event:** samplingProtocol: Entomological Net; verbatimEventDate: 7.v.2023; **Record Level:** collectionID: PNFCC000049
- **Type status:** Other material. **Occurrence:** sex: 2 males, 1 female; occurrenceID: FFEE1143-5132-50F3-9226-8BFDA3F670B2; **Taxon:** scientificName: Myathropaflorea (Linnaeus, 1758); order: Diptera; family: Syrphidae; **Location:** country: Italy; locality: PNFC Bucine (FC) 907m; decimalLatitude: 43.9594; decimalLongitude: 11.7097; geodeticDatum: WGS84; **Event:** samplingProtocol: Entomological Net; verbatimEventDate: 12.vi.2023; **Record Level:** collectionID: PNFCC000050, PNFCC000051, PNFCC000052
- **Type status:** Other material. **Occurrence:** sex: 1 male; occurrenceID: BC54D6B3-34E3-5686-A98F-85F5DDDD2429; **Taxon:** scientificName: Myathropaflorea (Linnaeus, 1758); order: Diptera; family: Syrphidae; **Location:** country: Italy; locality: PNFC Monte Falco (FI) 1653m; decimalLatitude: 43.8773; decimalLongitude: 11.7107; geodeticDatum: WGS84; **Event:** samplingProtocol: Entomological Net; verbatimEventDate: 11.viii.2023; **Record Level:** collectionID: PNFCC000053
- **Type status:** Other material. **Occurrence:** sex: 1 male, 1 female; occurrenceID: F628827A-C6B6-55D4-A2BA-E372C35EF219; **Taxon:** scientificName: Myathropaflorea (Linnaeus, 1758); order: Diptera; family: Syrphidae; **Location:** country: Italy; locality: PNFC Bucine (FC) 907m; decimalLatitude: 43.9594; decimalLongitude: 11.7097; geodeticDatum: WGS84; **Event:** samplingProtocol: Entomological Net; verbatimEventDate: 12.viii.2023; **Record Level:** collectionID: PNFCC000054

##### Notes

Recorded in Burgio et al. (2000).

#### Neoascia annexa

(Müller, 1776)

69BA06CB-7AD5-56AC-9FA8-1860F679351C

##### Notes

Recorded in Burgio et al. (2000).

#### Neoascia podagrica

(Fabricius, 1775)

4F9BADFE-D22B-596A-9CE4-2E67F206CB32

##### Notes

Recorded in Burgio et al. (2000).

#### Parasyrphus lineola

(Zetterstedt, 1843)

A7F04E46-9FCE-56D0-A9B8-A37B5F73900B

##### Notes

Recorded in Burgio et al. (2000)

#### Parasyrphus macularis

(Zetterstedt, 1843)

817665AB-AD1E-591E-B56E-295231E495F4

##### Notes

Recorded in Burgio et al. (2000).

#### Parasyrphus punctulatus

(Verrall, 1873)

0FEB04FA-135E-5098-8545-FC64C5A470B1

##### Materials

- **Type status:** Other material. **Occurrence:** sex: 1 female; occurrenceID: 8A82571C-8C45-53B1-A0F3-EB613222914A; **Taxon:** scientificName: Parasyrphuspunctulatus (Verrall, 1873); order: Diptera; family: Syrphidae; **Location:** country: Italy; locality: PNFC Foresta Lama Fonte Cavalla; decimalLatitude: 43.8238539; decimalLongitude: 11.8835054; geodeticDatum: WGS84; **Event:** samplingProtocol: Malaise Trap; verbatimEventDate: 18.v-8.vi.2004; **Record Level:** collectionID: AMSUB005059
- **Type status:** Other material. **Occurrence:** sex: 1 female; occurrenceID: 89F79066-8828-5181-84D8-A7C2C8EE8693; **Taxon:** scientificName: Parasyrphuspunctulatus (Verrall, 1873); order: Diptera; family: Syrphidae; **Location:** country: Italy; locality: PNFC Foresta Lama P. Saporita; decimalLatitude: 43.821774; decimalLongitude: 11.8802281; geodeticDatum: WGS84; **Event:** samplingProtocol: Malaise Trap; verbatimEventDate: 9.vii.2004; **Record Level:** collectionID: AMSUB005060

##### Notes

Recorded in Burgio et al. (2000).

#### Pipiza festiva

Meigen, 1822

1D44DD48-BFA4-5487-BE39-D195E748843B

##### Materials

- **Type status:** Other material. **Occurrence:** sex: 1 female; occurrenceID: D7CFACB7-DD73-5B1D-A556-C4B3E3FD2A34; **Taxon:** scientificName: Pipizafestiva Meigen, 1822; order: Diptera; family: Syrphidae; **Location:** country: Italy; locality: PNFC Palazzuolo sul Senio (FI) 400 m; decimalLatitude: 44.1135; decimalLongitude: 11.5468; geodeticDatum: WGS84; **Event:** samplingProtocol: Entomological Net; verbatimEventDate: ix.91; **Record Level:** collectionID: AMSUB005345

#### Pipiza noctiluca

(Linnaeus, 1758)

4E17F5EC-2B63-5E85-B3D8-0BAC6AA41DEB

##### Materials

- **Type status:** Other material. **Occurrence:** sex: 1 female; occurrenceID: CE1655CB-DED3-59C7-8A6D-04A7BF098F1B; **Taxon:** scientificName: Pipizanoctiluca (Linnaeus, 1758); order: Diptera; family: Syrphidae; **Location:** country: Italy; locality: PNFC Foresta Lama Fonte Cavalla; decimalLatitude: 43.8238539; decimalLongitude: 11.8835054; geodeticDatum: WGS84; **Event:** samplingProtocol: Malaise Trap; verbatimEventDate: 18.v-8.vi.2004; **Record Level:** collectionID: AMSUB005058

#### Pipizella annulata

(Macquart, 1829)

3FF7BAE8-F26A-576C-8E77-839F7B7C1932

##### Notes

Recorded in Burgio et al. (2000).

#### Pipizella elegantissima

Lucas, 1976

5AC025E1-3802-5FEA-B319-B8732EA89379

##### Materials

- **Type status:** Other material. **Occurrence:** sex: 1 male; occurrenceID: 1202C56F-FEC2-5779-8BC6-89FB39205C95; **Taxon:** scientificName: Pipizellaelegantissima Lucas, 1976; order: Diptera; family: Syrphidae; **Location:** country: Italy; locality: PNFC Burraia (FC) m. 1500; decimalLatitude: 43.8657; decimalLongitude: 11.7347; geodeticDatum: WGS84; **Event:** samplingProtocol: Entomological Net; verbatimEventDate: 16.vii.1994; **Record Level:** collectionID: AMSUB005352

##### Notes

Recorded in Burgio et al. (2000).

#### Platycheirus albimanus

(Fabricius, 1781)

A2D2AF7A-914B-59A3-BC27-2B1F73CB0016

##### Materials

- **Type status:** Other material. **Occurrence:** sex: 1 male; occurrenceID: 8B5B8648-0898-5CBB-A1F9-6E2A09985E38; **Taxon:** scientificName: Platycheirusalbimanus (Fabricius, 1781); order: Diptera; family: Syrphidae; **Location:** country: Italy; locality: PNFC Foresta Lama Fonte Cavalla; decimalLatitude: 43.8238539; decimalLongitude: 11.8835054; geodeticDatum: WGS84; **Event:** samplingProtocol: Malaise Trap; verbatimEventDate: 18.v-8.vi.2004; **Record Level:** collectionID: AMSUB005054
- **Type status:** Other material. **Occurrence:** sex: 1 male; occurrenceID: 1DE6F155-9097-5456-A5E8-77D000A4B5FC; **Taxon:** scientificName: Platycheirusalbimanus (Fabricius, 1781); order: Diptera; family: Syrphidae; **Location:** country: Italy; locality: PNFC Foresta Lama P. Saporita; decimalLatitude: 43.821774; decimalLongitude: 11.8802281; geodeticDatum: WGS84; **Event:** samplingProtocol: Malaise Trap; verbatimEventDate: 27.vii.2004; **Record Level:** collectionID: AMSUB005127
- **Type status:** Other material. **Occurrence:** sex: 1 male; occurrenceID: DA4755AD-E671-57F5-8203-FF18DF1AF3D0; **Taxon:** scientificName: Platycheirusalbimanus (Fabricius, 1781); order: Diptera; family: Syrphidae; **Location:** country: Italy; locality: PNFC Foresta Lama Stazione Prati; decimalLatitude: 43.8313707; decimalLongitude: 11.8380049; geodeticDatum: WGS84; **Event:** samplingProtocol: Malaise Trap; verbatimEventDate: 3-14.vii.2003; **Record Level:** collectionID: AMSUB005285
- **Type status:** Other material. **Occurrence:** sex: 5 females; occurrenceID: CB543B58-8483-5EA5-81E4-B7C5669C3B26; **Taxon:** scientificName: Platycheirusalbimanus (Fabricius, 1781); order: Diptera; family: Syrphidae; **Location:** country: Italy; locality: PNFC Foresta Lama Stazione Prati; decimalLatitude: 43.8313707; decimalLongitude: 11.8380049; geodeticDatum: WGS84; **Event:** samplingProtocol: Malaise Trap; verbatimEventDate: 6-16.viii.2003; **Record Level:** collectionID: AMSUB005305, AMSUB005306, AMSUB005307, AMSUB005308, AMSUB005309
- **Type status:** Other material. **Occurrence:** sex: 20 females; occurrenceID: D506C1CC-4BC9-54EF-A628-A9769C2E36B3; **Taxon:** scientificName: Platycheirusalbimanus (Fabricius, 1781); order: Diptera; family: Syrphidae; **Location:** country: Italy; locality: PNFC Monte Falco (FI) 1653m; decimalLatitude: 43.8773; decimalLongitude: 11.7107; geodeticDatum: WGS84; **Event:** samplingProtocol: Entomological Net; verbatimEventDate: 6.v.2023; **Record Level:** collectionID: PNFCC000056, PNFCC000057, PNFCC000058, PNFCC000059, PNFCC000060, PNFCC000061, PNFCC000062, PNFCC000063, PNFCC000064, PNFCC000065, PNFCC000066, PNFCC000067, PNFCC000068, PNFCC000069, PNFCC000070, PNFCC000071, PNFCC000072, PNFCC000073, PNFCC000074, PNFCC000075
- **Type status:** Other material. **Occurrence:** sex: 1 females; occurrenceID: EC5E1E69-74B8-51AA-A06D-61E9818D5B77; **Taxon:** scientificName: Platycheirusalbimanus (Fabricius, 1781); order: Diptera; family: Syrphidae; **Location:** country: Italy; locality: PNFC Monte Falco (FI) 1653m; decimalLatitude: 43.8773; decimalLongitude: 11.7107; geodeticDatum: WGS84; **Event:** samplingProtocol: Entomological Net; verbatimEventDate: 11.vi.2023; **Record Level:** collectionID: PNFCC000076, PNFCC000077, PNFCC000078, PNFCC000079, PNFCC000080, PNFCC000081, PNFCC000082
- **Type status:** Other material. **Occurrence:** sex: 2 exx; occurrenceID: 1C19EFFF-E1EF-55AB-BADE-B19C76548B00; **Taxon:** scientificName: Platycheirusalbimanus (Fabricius, 1781); order: Diptera; family: Syrphidae; **Location:** country: Italy; locality: PNFC Monte Falco (FI) 1653m; decimalLatitude: 43.8773; decimalLongitude: 11.7107; geodeticDatum: WGS84; **Event:** samplingProtocol: Observation; verbatimEventDate: 11.vi.2023
- **Type status:** Other material. **Occurrence:** sex: 1 female; occurrenceID: 25C2557C-BAF0-5E33-BFD8-DB82F03BB0D1; **Taxon:** scientificName: Platycheirusalbimanus (Fabricius, 1781); order: Diptera; family: Syrphidae; **Location:** country: Italy; locality: PNFC Bucine (FC) 907m; decimalLatitude: 43.9594; decimalLongitude: 11.7097; geodeticDatum: WGS84; **Event:** samplingProtocol: Entomological Net; verbatimEventDate: 12.vi.2023; **Record Level:** collectionID: PNFCC000083
- **Type status:** Other material. **Occurrence:** sex: 1 male; occurrenceID: 5607C70D-4EAA-58FE-B317-F6D9A25CBF04; **Taxon:** scientificName: Platycheirusalbimanus (Fabricius, 1781); order: Diptera; family: Syrphidae; **Location:** country: Italy; locality: PNFC Monte Falco (FI) 1653m; decimalLatitude: 43.8773; decimalLongitude: 11.7107; geodeticDatum: WGS84; **Event:** samplingProtocol: Entomological Net; verbatimEventDate: 11.viii.2023; **Record Level:** collectionID: PNFCC000084

##### Notes

Recorded in Burgio et al. (2000).

#### Platycheirus fulviventris

(Macquart, 1829)

E44D1E14-FB48-56E7-A40C-F2BCAF44B2CA

##### Materials

- **Type status:** Other material. **Occurrence:** sex: 1 female; occurrenceID: 9451E68A-6179-50A0-ACB9-287D1ECEB1C5; **Taxon:** scientificName: Platycheirusfulviventris (Macquart, 1829); order: Diptera; family: Syrphidae; **Location:** country: Italy; locality: PNFC Foresta Lama P. Saporita; decimalLatitude: 43.821774; decimalLongitude: 11.8802281; geodeticDatum: WGS84; **Event:** samplingProtocol: Malaise Trap; verbatimEventDate: 9.vii.2004; **Record Level:** collectionID: AMSUB005085

#### Platycheirus immaculatus

Ohara, 1980

8A13BD01-A3B7-526C-9CD7-3F6952D8899D

##### Materials

- **Type status:** Other material. **Occurrence:** sex: 1 male; occurrenceID: 7E697C38-76D1-5ECE-A68E-1CE9E492F92B; **Taxon:** scientificName: Platycheirusimmaculatus Ohara, 1980; order: Diptera; family: Syrphidae; **Location:** country: Italy; locality: PNFC Foresta Lama Stazione Vetreria; decimalLatitude: 43.829961; decimalLongitude: 11.8418409; geodeticDatum: WGS84; **Event:** samplingProtocol: Malaise Trap; verbatimEventDate: 26.v-6.vi.2003; **Record Level:** collectionID: AMSUB005188

#### Platycheirus scutatus

(Meigen, 1822)

B023C5A5-A20C-5894-9495-2E652ECF49BE

##### Materials

- **Type status:** Other material. **Occurrence:** sex: 1 female; occurrenceID: 0B407DFC-9F7E-55F9-BA94-468F80E5FCE0; **Taxon:** scientificName: Platycheirusscutatus (Meigen, 1822); order: Diptera; family: Syrphidae; **Location:** country: Italy; locality: PNFC Foresta Lama Bertesca; decimalLatitude: 43.8314943; decimalLongitude: 11.8735824; geodeticDatum: WGS84; **Event:** samplingProtocol: Malaise Trap; verbatimEventDate: 30.vi.2004; **Record Level:** collectionID: AMSUB005129
- **Type status:** Other material. **Occurrence:** sex: 1 female; occurrenceID: CB6B6C03-2DF7-510A-BCD5-7C1EB3AFFA22; **Taxon:** scientificName: Platycheirusscutatus (Meigen, 1822); order: Diptera; family: Syrphidae; **Location:** country: Italy; locality: PNFC Foresta Lama Bertesca; decimalLatitude: 43.8314943; decimalLongitude: 11.8735824; geodeticDatum: WGS84; **Event:** samplingProtocol: Malaise Trap; verbatimEventDate: 27.vii.2004; **Record Level:** collectionID: AMSUB005173
- **Type status:** Other material. **Occurrence:** sex: 2 males; occurrenceID: 8BFD314D-C273-53E2-BFD8-FCBDC7F038B2; **Taxon:** scientificName: Platycheirusscutatus (Meigen, 1822); order: Diptera; family: Syrphidae; **Location:** country: Italy; locality: PNFC Bucine (FC) 907m; decimalLatitude: 43.9495; decimalLongitude: 11.7097; geodeticDatum: WGS84; **Event:** samplingProtocol: Entomological Net; verbatimEventDate: 7.v.2023; **Record Level:** collectionID: PNFCC000085, PNFCC000086

##### Notes

Recorded in Burgio et al. (2000).

#### Rhingia rostrata

(Linnaeus, 1758)

6E0E26EF-CA10-5272-8637-0277821C45A8

##### Materials

- **Type status:** Other material. **Occurrence:** sex: 1 female; occurrenceID: 9EFEBA0E-85BA-5D2D-8765-727FE401B8A7; **Taxon:** scientificName: Rhingiarostrata (Linnaeus, 1758); order: Diptera; family: Syrphidae; **Location:** country: Italy; locality: PNFC Foresta Lama Stazione Prati; decimalLatitude: 43.8313707; decimalLongitude: 11.8380049; geodeticDatum: WGS84; **Event:** samplingProtocol: Malaise Trap; verbatimEventDate: 26.v-6.vi.2003; **Record Level:** collectionID: AMSUB005194

#### Scaeva pyrastri

(Linnaeus, 1758)

6FF00AC9-3358-5B66-90C4-9C66E7919537

##### Materials

- **Type status:** Other material. **Occurrence:** sex: 1 female; occurrenceID: 8D9D46D9-DB84-5049-8642-E3AC541B0D2E; **Taxon:** scientificName: Scaevapyrastri (Linnaeus, 1758); order: Diptera; family: Syrphidae; **Location:** country: Italy; locality: PNFC Foresta Lama Bertesca; decimalLatitude: 43.8314943; decimalLongitude: 11.8735824; geodeticDatum: WGS84; **Event:** samplingProtocol: Malaise Trap; verbatimEventDate: 27.vii.2004; **Record Level:** collectionID: AMSUB005162

##### Notes

Recorded in Burgio et al. (2000).

#### Scaeva selenitica

(Meigen, 1822)

AC947AED-D884-568B-82C7-996904FD77F6

##### Notes

Recorded in Burgio et al. (2000).

#### Sericomyia bombiformis

(Fallén, 1810)

556A0E4A-5088-5FA8-A3A9-3B593FB7B3CC

##### Notes

Recorded in Burgio et al. (2000).

#### Sericomyia superbiens

(Müller, 1776)

C7FDA4DB-2426-5DE8-9552-BA5D8106C982

##### Materials

- **Type status:** Other material. **Occurrence:** sex: 1 male; occurrenceID: D6F26FF6-76E8-5CE9-8152-DA9CE8650473; **Taxon:** scientificName: Sericomyiasuperbiens (Müller, 1776); order: Diptera; family: Syrphidae; **Location:** country: Italy; locality: PNFC Piedimonte (FI) m. 500; decimalLatitude: 44.0924; decimalLongitude: 11.5185; geodeticDatum: WGS84; **Event:** samplingProtocol: Entomological Net; verbatimEventDate: 20.viii.1994; **Record Level:** collectionID: AMSUB005328

##### Notes

Recorded in Burgio et al. (2000).

#### Sphaerophoria laurae

Goeldlin, 1989

F150EC65-C49A-5F9F-A899-BF9819CC41F3

##### Materials

- **Type status:** Other material. **Occurrence:** sex: 1 male; occurrenceID: 346F0829-DACE-5F5D-9EAC-DB8D6ABBF73C; **Taxon:** scientificName: Sphaerophorialaurae Goeldlin, 1989; order: Diptera; family: Syrphidae; **Location:** country: Italy; locality: PNFC Foresta Lama Stazione Prati; decimalLatitude: 43.8313707; decimalLongitude: 11.8380049; geodeticDatum: WGS84; **Event:** samplingProtocol: Malaise Trap; verbatimEventDate: 3-14.vii.2003; **Record Level:** collectionID: AMSUB005278

#### Sphaerophoria scripta

(Linnaeus, 1758)

61F07AE0-18E3-5108-8F83-8463E08DE4DC

##### Materials

- **Type status:** Other material. **Occurrence:** sex: 3 males, 4 females; occurrenceID: A68FEBBA-D616-599C-9817-C58D2A37FFAC; **Taxon:** scientificName: Sphaerophoriascripta (Linnaeus, 1758); order: Diptera; family: Syrphidae; **Location:** country: Italy; locality: PNFC Foresta Lama Fonte Cavalla; decimalLatitude: 43.8238539; decimalLongitude: 11.8835054; geodeticDatum: WGS84; **Event:** samplingProtocol: Malaise Trap; verbatimEventDate: 18.v-8.vi.2004; **Record Level:** collectionID: AMSUB005006, AMSUB005007, AMSUB005008, AMSUB005017, AMSUB005018, AMSUB005019, AMSUB005020
- **Type status:** Other material. **Occurrence:** sex: 2 males, 1 female; occurrenceID: 2C6D4AC1-FE7E-516E-8668-C49FF5CBB142; **Taxon:** scientificName: Sphaerophoriascripta (Linnaeus, 1758); order: Diptera; family: Syrphidae; **Location:** country: Italy; locality: PNFC Foresta Lama P. Saporita; decimalLatitude: 43.821774; decimalLongitude: 11.8802281; geodeticDatum: WGS84; **Event:** samplingProtocol: Malaise Trap; verbatimEventDate: 27.vii.2004; **Record Level:** collectionID: AMSUB005025, AMSUB005026, AMSUB005100
- **Type status:** Other material. **Occurrence:** sex: 1 male; occurrenceID: CBFD5A8D-9DE5-5884-9290-88AF832E4AD8; **Taxon:** scientificName: Sphaerophoriascripta (Linnaeus, 1758); order: Diptera; family: Syrphidae; **Location:** country: Italy; locality: PNFC Foresta Lama Fonte Cavalla; decimalLatitude: 43.8238539; decimalLongitude: 11.8835054; geodeticDatum: WGS84; **Event:** samplingProtocol: Malaise Trap; verbatimEventDate: 18.v-8.vi.2004; **Record Level:** collectionID: AMSUB005053
- **Type status:** Other material. **Occurrence:** sex: 1 male; occurrenceID: 9083BA6C-3195-5972-8F98-919DC021D654; **Taxon:** scientificName: Sphaerophoriascripta (Linnaeus, 1758); order: Diptera; family: Syrphidae; **Location:** country: Italy; locality: PNFC Foresta Lama P. Saporita; decimalLatitude: 43.821774; decimalLongitude: 11.8802281; geodeticDatum: WGS84; **Event:** samplingProtocol: Malaise Trap; verbatimEventDate: 9.vii.2004; **Record Level:** collectionID: AMSUB005082
- **Type status:** Other material. **Occurrence:** sex: 1 male; occurrenceID: ED347E81-7BAF-533C-B8D5-558B306CA33B; **Taxon:** scientificName: Sphaerophoriascripta (Linnaeus, 1758); order: Diptera; family: Syrphidae; **Location:** country: Italy; locality: PNFC Foresta Lama Bertesca; decimalLatitude: 43.8314943; decimalLongitude: 11.8735824; geodeticDatum: WGS84; **Event:** samplingProtocol: Malaise Trap; verbatimEventDate: 30.vi.2004; **Record Level:** collectionID: AMSUB005139
- **Type status:** Other material. **Occurrence:** sex: 1 male; occurrenceID: 36ADB1D2-BF9A-57C8-9D8F-1FE0D0B14C36; **Taxon:** scientificName: Sphaerophoriascripta (Linnaeus, 1758); order: Diptera; family: Syrphidae; **Location:** country: Italy; locality: PNFC Foresta Lama Bertesca; decimalLatitude: 43.8314943; decimalLongitude: 11.8735824; geodeticDatum: WGS84; **Event:** samplingProtocol: Malaise Trap; verbatimEventDate: 27.vii.2004; **Record Level:** collectionID: AMSUB005163
- **Type status:** Other material. **Occurrence:** sex: 2 females; occurrenceID: E593D23D-0335-5845-BDDD-709226977B7F; **Taxon:** scientificName: Sphaerophoriascripta (Linnaeus, 1758); order: Diptera; family: Syrphidae; **Location:** country: Italy; locality: PNFC Foresta Lama Stazione Prati; decimalLatitude: 43.8313707; decimalLongitude: 11.8380049; geodeticDatum: WGS84; **Event:** samplingProtocol: Malaise Trap; verbatimEventDate: 3-14.vii.2003; **Record Level:** collectionID: AMSUB005279, AMSUB005280
- **Type status:** Other material. **Occurrence:** sex: 1 female; occurrenceID: 27228BFE-42D7-5032-9BBA-D82E1AECA5A8; **Taxon:** scientificName: Sphaerophoriascripta (Linnaeus, 1758); order: Diptera; family: Syrphidae; **Location:** country: Italy; locality: PNFC Foresta Lama Vetreria; decimalLatitude: 43.829961; decimalLongitude: 11.8380049; geodeticDatum: WGS84; **Event:** samplingProtocol: Malaise Trap; verbatimEventDate: 3-14.vii.2003; **Record Level:** collectionID: AMSUB005327
- **Type status:** Other material. **Occurrence:** sex: 1 female; occurrenceID: 1CE211B5-7670-531A-BECB-BB5B04460CFC; **Taxon:** scientificName: Sphaerophoriascripta (Linnaeus, 1758); order: Diptera; family: Syrphidae; **Location:** country: Italy; locality: PNFC Fangacci (FC) 1400; decimalLatitude: 43.8103; decimalLongitude: 11.8489; geodeticDatum: WGS84; **Event:** samplingProtocol: Entomological Net; verbatimEventDate: 9.v.1994; **Record Level:** collectionID: AMSUB005341
- **Type status:** Other material. **Occurrence:** sex: 1 female; occurrenceID: 02CA1AEF-D010-5AC3-936F-04D8D9806EF2; **Taxon:** scientificName: Sphaerophoriascripta (Linnaeus, 1758); order: Diptera; family: Syrphidae; **Location:** country: Italy; locality: PNFC La stretta (FC); **Event:** samplingProtocol: Entomological Net; verbatimEventDate: 8.vii.1994; **Record Level:** collectionID: AMSUB005342
- **Type status:** Other material. **Occurrence:** sex: 3 males, 1 female; occurrenceID: B53FB91E-E7F8-5648-A891-66FFF242F4BF; **Taxon:** scientificName: Sphaerophoriascripta (Linnaeus, 1758); order: Diptera; family: Syrphidae; **Location:** country: Italy; locality: PNFC Bucine (FC) 907m; decimalLatitude: 43.9594; decimalLongitude: 11.7097; geodeticDatum: WGS84; **Event:** samplingProtocol: Entomological Net; verbatimEventDate: 7.v.2023; **Record Level:** collectionID: PNFCC000087, PNFCC000088, PNFCC000089, PNFCC000090
- **Type status:** Other material. **Occurrence:** sex: 2 females; occurrenceID: 79580377-D207-50C8-949F-7A6A6C99B25E; **Taxon:** scientificName: Sphaerophoriascripta (Linnaeus, 1758); order: Diptera; family: Syrphidae; **Location:** country: Italy; locality: PNFC Monte Falco (FI) 1653m; decimalLatitude: 43.8773; decimalLongitude: 11.7107; geodeticDatum: WGS84; **Event:** samplingProtocol: Entomological Net; verbatimEventDate: 11.vi.2023; **Record Level:** collectionID: PNFCC000091, PNFCC000092
- **Type status:** Other material. **Occurrence:** sex: 2 males, 6 females; occurrenceID: F5206453-B631-5544-89B2-A76159C0E4B2; **Taxon:** scientificName: Sphaerophoriascripta (Linnaeus, 1758); order: Diptera; family: Syrphidae; **Location:** country: Italy; locality: PNFC Bucine (FC) 907m; decimalLatitude: 43.9594; decimalLongitude: 11.7097; geodeticDatum: WGS84; **Event:** samplingProtocol: Entomological Net; verbatimEventDate: 12.vi.2023; **Record Level:** collectionID: PNFCC000093, PNFCC000094, PNFCC000095, PNFCC000096, PNFCC000097, PNFCC000098, PNFCC000099, PNFCC000100
- **Type status:** Other material. **Occurrence:** sex: 2 males, 1 female; occurrenceID: CF92CC6E-D795-58DC-90E0-90FD64D9E04A; **Taxon:** scientificName: Sphaerophoriascripta (Linnaeus, 1758); order: Diptera; family: Syrphidae; **Location:** country: Italy; locality: PNFC Monte Falco (FI) 1653m; decimalLatitude: 43.8773; decimalLongitude: 11.7107; geodeticDatum: WGS84; **Event:** samplingProtocol: Entomological Net; verbatimEventDate: 11.vii.2023; **Record Level:** collectionID: PNFCC000101, PNFCC000102, PNFCC000103
- **Type status:** Other material. **Occurrence:** sex: 7 males, 2 females; occurrenceID: C6B4AA44-1E40-5AC6-A94A-7365DEC5CB3A; **Taxon:** scientificName: Sphaerophoriascripta (Linnaeus, 1758); order: Diptera; family: Syrphidae; **Location:** country: Italy; locality: PNFC Bucine (FC) 907m; decimalLatitude: 43.9594; decimalLongitude: 11.7097; geodeticDatum: WGS84; **Event:** samplingProtocol: Entomological Net; verbatimEventDate: 12.vii.2023; **Record Level:** collectionID: PNFCC000104, PNFCC000105, PNFCC000106, PNFCC000107, PNFCC000108, PNFCC000109, PNFCC000110, PNFCC000111, PNFCC000112
- **Type status:** Other material. **Occurrence:** sex: 2 females; occurrenceID: 4D7D4055-25AA-5860-B1FF-5BBD33054144; **Taxon:** scientificName: Sphaerophoriascripta (Linnaeus, 1758); order: Diptera; family: Syrphidae; **Location:** country: Italy; locality: PNFC Bucine (FC) 907m; decimalLatitude: 43.9594; decimalLongitude: 11.7097; geodeticDatum: WGS84; **Event:** samplingProtocol: Entomological Net; verbatimEventDate: 12.viii.2023; **Record Level:** collectionID: PNFCC000113, PNFCC000114

##### Notes

Recorded in Burgio et al. (2000).

#### Sphegina clunipes

(Fallén, 1816)

FAE826D4-E6B4-5155-8D65-ADCFE01CFC78

##### Notes

Recorded in Burgio et al. (2000).

#### Sphegina verecunda

Collin, 1937

17144A1B-C1B7-50F1-8D3F-6B6CB27FB4BA

##### Notes

Recorded in Burgio et al. (2000).

#### Syritta pipiens

(Linnaeus, 1758)

3C3E95C5-36DA-561C-80DD-09E449C83EC9

##### Notes

Recorded in Burgio et al. (2000).

#### Syrphus nitidifrons

Becker, 1921

1817FB76-5B4C-5B66-89FA-D23E7429A62D

##### Notes

Recorded in Burgio et al. (2000).

#### Syrphus ribesii

(Linnaeus, 1758)

52F2EB50-723A-5233-9FB8-F85B60A814B1

##### Materials

- **Type status:** Other material. **Occurrence:** sex: 1 male; occurrenceID: DA88A577-0AD6-5EE8-A120-B9BC070909C4; **Taxon:** scientificName: Syrphusribesii (Linnaeus, 1758); order: Diptera; family: Syrphidae; **Location:** country: Italy; locality: PNFC Foresta Lama P. Saporita; decimalLatitude: 43.821774; decimalLongitude: 11.8802281; geodeticDatum: WGS84; **Event:** samplingProtocol: Malaise Trap; verbatimEventDate: 27.vii.2004; **Record Level:** collectionID: AMSUB005122
- **Type status:** Other material. **Occurrence:** sex: 1 female; occurrenceID: CB4C508B-0EF5-5114-9452-1F35272D71E2; **Taxon:** scientificName: Syrphusribesii (Linnaeus, 1758); order: Diptera; family: Syrphidae; **Location:** country: Italy; locality: PNFC Foresta Lama Bertesca; decimalLatitude: 43.8314943; decimalLongitude: 11.8735824; geodeticDatum: WGS84; **Event:** samplingProtocol: Malaise Trap; verbatimEventDate: 27.vii.2004; **Record Level:** collectionID: AMSUB005159
- **Type status:** Other material. **Occurrence:** sex: 1 female; occurrenceID: B31DAAFF-0B9B-58A7-A674-F84E93E98F8C; **Taxon:** scientificName: Syrphusribesii (Linnaeus, 1758); order: Diptera; family: Syrphidae; **Location:** country: Italy; locality: PNFC Fangacci (FC) 1400; decimalLatitude: 43.8103; decimalLongitude: 11.8489; geodeticDatum: WGS84; **Event:** samplingProtocol: Entomological Net; verbatimEventDate: 23.vii.1994; **Record Level:** collectionID: AMSUB005343
- **Type status:** Other material. **Occurrence:** sex: 1 male; occurrenceID: 23E8404F-F211-551F-8AB0-6DCED938E3B7; **Taxon:** scientificName: Syrphusribesii (Linnaeus, 1758); order: Diptera; family: Syrphidae; **Location:** country: Italy; locality: PNFC Bucine (FC) 907m; decimalLatitude: 43.9594; decimalLongitude: 11.7097; geodeticDatum: WGS84; **Event:** samplingProtocol: Entomological Net; verbatimEventDate: 12.vi.2023; **Record Level:** collectionID: PNFCC000115

##### Notes

Recorded in Burgio et al. (2000).

#### Syrphus torvus

Osten-sacken, 1875

BC0B0F1E-C35E-5AB4-BF4A-B7FB907A87AC

##### Materials

- **Type status:** Other material. **Occurrence:** sex: 1 male; occurrenceID: F2A0D0CF-7A61-57F0-9217-70B712EEC8F2; **Taxon:** scientificName: Syrphustorvus Osten-sacken, 1875; order: Diptera; family: Syrphidae; **Location:** country: Italy; locality: PNFC Fangacci (FC) 1400; decimalLatitude: 43.8103; decimalLongitude: 11.8489; geodeticDatum: WGS84; **Event:** samplingProtocol: Entomological Net; verbatimEventDate: 23.vii.1994; **Record Level:** collectionID: AMSUB005343
- **Type status:** Other material. **Occurrence:** sex: 3 females; occurrenceID: E06BA5E4-6ADD-54EE-B52C-C400E954B680; **Taxon:** scientificName: Syrphustorvus Osten-sacken, 1875; order: Diptera; family: Syrphidae; **Location:** country: Italy; locality: PNFC Monte Falco (FI) 1653m; decimalLatitude: 43.8773; decimalLongitude: 11.7107; geodeticDatum: WGS84; **Event:** samplingProtocol: Entomological Net; verbatimEventDate: 6.v.2023; **Record Level:** collectionID: PNFCC000116, PNFCC000117, PNFCC000118
- **Type status:** Other material. **Occurrence:** sex: 1 male; occurrenceID: 2AD1076E-44D3-5697-9B1F-009CA6205525; **Taxon:** scientificName: Syrphustorvus Osten-sacken, 1875; order: Diptera; family: Syrphidae; **Location:** country: Italy; locality: PNFC Monte Falco (FI) 1653m; decimalLatitude: 43.8773; decimalLongitude: 11.7107; geodeticDatum: WGS84; **Event:** samplingProtocol: Entomological Net; verbatimEventDate: 11.vi.2023; **Record Level:** collectionID: PNFCC000119
- **Type status:** Other material. **Occurrence:** sex: 6 males, 3 females; occurrenceID: 7BD5759A-D9A0-5B35-8026-598964C14CE8; **Taxon:** scientificName: Syrphustorvus Osten-sacken, 1875; order: Diptera; family: Syrphidae; **Location:** country: Italy; locality: PNFC Bucine (FC) 907m; decimalLatitude: 43.9594; decimalLongitude: 11.7097; geodeticDatum: WGS84; **Event:** samplingProtocol: Entomological Net; verbatimEventDate: 12.vi.2023; **Record Level:** collectionID: PNFCC000120, PNFCC000121, PNFCC000122, PNFCC000123, PNFCC000124, PNFCC000125, PNFCC000126, PNFCC000127, PNFCC000128
- **Type status:** Other material. **Occurrence:** sex: 1 male, 1 female; occurrenceID: 07625D28-3F2D-5BCD-A3B6-4E2E7EA68CAE; **Taxon:** scientificName: Syrphustorvus Osten-sacken, 1875; order: Diptera; family: Syrphidae; **Location:** country: Italy; locality: PNFC Monte Falco (FI) 1653m; decimalLatitude: 43.8773; decimalLongitude: 11.7107; geodeticDatum: WGS84; **Event:** samplingProtocol: Entomological Net; verbatimEventDate: 11.viii.2023; **Record Level:** collectionID: PNFCC000129, PNFCC000130

#### Syrphus vitripennis

Meigen, 1822

7C7A4C58-378F-57E7-825D-2908D1E597F1

##### Materials

- **Type status:** Other material. **Occurrence:** sex: 2 males; occurrenceID: E229CFE2-25F7-57E4-87EC-F4514BD361CB; **Taxon:** scientificName: Syrphusvitripennis Meigen, 1822; order: Diptera; family: Syrphidae; **Location:** country: Italy; locality: PNFC Foresta Lama P. Saporita; decimalLatitude: 43.821774; decimalLongitude: 11.8802281; geodeticDatum: WGS84; **Event:** samplingProtocol: Malaise Trap; verbatimEventDate: 27.vii.2004; **Record Level:** collectionID: AMSUB005123, AMSUB005124
- **Type status:** Other material. **Occurrence:** sex: 17 males, 11 females; occurrenceID: 29C343AD-88CA-5CE5-BE61-B72764F1EDA6; **Taxon:** scientificName: Syrphusvitripennis Meigen, 1822; order: Diptera; family: Syrphidae; **Location:** country: Italy; locality: PNFC Foresta Lama Stazione Prati; decimalLatitude: 43.8313707; decimalLongitude: 11.8380049; geodeticDatum: WGS84; **Event:** samplingProtocol: Malaise Trap; verbatimEventDate: 3-14.vii.2003; **Record Level:** collectionID: AMSUB005242, AMSUB005243, AMSUB005244, AMSUB005245, AMSUB005246, AMSUB005247, AMSUB005248, AMSUB005249, AMSUB005250, AMSUB005251, AMSUB005252, AMSUB005253, AMSUB005254, AMSUB005255, AMSUB005256, AMSUB005257, AMSUB005258, AMSUB005259, AMSUB005260, AMSUB005261, AMSUB005262, AMSUB005263, AMSUB005264, AMSUB005265, AMSUB005266,AMSUB005267, AMSUB005268, AMSUB005282
- **Type status:** Other material. **Occurrence:** sex: 5 females; occurrenceID: 197D79F4-92CB-5565-8945-E3CA2B480627; **Taxon:** scientificName: Syrphusvitripennis Meigen, 1822; order: Diptera; family: Syrphidae; **Location:** country: Italy; locality: PNFC Foresta Lama Stazione Prati; decimalLatitude: 43.8313707; decimalLongitude: 11.8380049; geodeticDatum: WGS84; **Event:** samplingProtocol: Malaise Trap; verbatimEventDate: 6-16.viii.2003; **Record Level:** collectionID: AMSUB005310, AMSUB005311, AMSUB005312, AMSUB005313, AMSUB005314
- **Type status:** Other material. **Occurrence:** sex: 1 male; occurrenceID: CF1748BB-CEE6-5F4F-BAA9-04C6E0FD4017; **Taxon:** scientificName: Syrphusvitripennis Meigen, 1822; order: Diptera; family: Syrphidae; **Location:** country: Italy; locality: PNFC Monte Falco (FI) 1653m; decimalLatitude: 43.8773; decimalLongitude: 11.7107; geodeticDatum: WGS84; **Event:** samplingProtocol: Entomological Net; verbatimEventDate: 6.v.2023; **Record Level:** collectionID: PNFCC000131
- **Type status:** Other material. **Occurrence:** sex: 5 males, 3 females; occurrenceID: 935F039E-D2DB-5DB4-BA6A-B99B5A5E8752; **Taxon:** scientificName: Syrphusvitripennis Meigen, 1822; order: Diptera; family: Syrphidae; **Location:** country: Italy; locality: PNFC Bucine (FC) 907m; decimalLatitude: 43.9594; decimalLongitude: 11.7097; geodeticDatum: WGS84; **Event:** samplingProtocol: Entomological Net; verbatimEventDate: 12.vi.2023; **Record Level:** collectionID: PNFCC000132, PNFCC000133, PNFCC000134, PNFCC000135, PNFCC000136, PNFCC000137, PNFCC000138, PNFCC000139, PNFCC000140
- **Type status:** Other material. **Occurrence:** sex: 1 female; occurrenceID: 78AB8524-01F6-5BFE-8173-2DD2E933D785; **Taxon:** scientificName: Syrphusvitripennis Meigen, 1822; order: Diptera; family: Syrphidae; **Location:** country: Italy; locality: PNFC Bucine (FC) 907m; decimalLatitude: 43.9594; decimalLongitude: 11.7097; geodeticDatum: WGS84; **Event:** samplingProtocol: Entomological Net; verbatimEventDate: 12.vii.2023; **Record Level:** collectionID: PNFCC000141
- **Type status:** Other material. **Occurrence:** sex: 1 male, 2 females; occurrenceID: 8F23F0A2-C4B0-599F-957A-1B9AEB69C568; **Taxon:** scientificName: Syrphusvitripennis Meigen, 1822; order: Diptera; family: Syrphidae; **Location:** country: Italy; locality: PNFC Monte Falco (FI) 1653m; decimalLatitude: 43.8773; decimalLongitude: 11.7107; geodeticDatum: WGS84; **Event:** samplingProtocol: Entomological Net; verbatimEventDate: 11.viii.2023; **Record Level:** collectionID: PNFCC000142, PNFCC000143, PNFCC000144

##### Notes

Recorded in Burgio et al. (2000).

#### Volucella bombylans

(Linnaeus, 1758)

D5B9F5F3-E8D0-51D1-B0E5-B76ACCB4381E

##### Notes

Recorded in Burgio et al. (2000).

#### Volucella inanis

(Linnaeus, 1758)

501F9196-77FD-5C8D-96E4-6A202B9E1FDE

##### Notes

Recorded in Burgio et al. (2000).

#### Volucella inflata

(Fabricius, 1794)

7F894678-199A-5F2D-A0F2-F183D75E2C4C

##### Notes

Recorded in Burgio et al. (2000).

#### Volucella pellucens

(Linnaeus, 1758)

C4B07703-4B33-598E-AA68-75BD25C7CB8B

##### Materials

- **Type status:** Other material. **Occurrence:** sex: 2 exx; occurrenceID: 0B5E7EBB-28AA-54C4-9283-2E36952A9E13; **Taxon:** scientificName: Volucellapellucens (Linnaeus, 1758); order: Diptera; family: Syrphidae; **Location:** country: Italy; locality: PNFC Monte Falco (FI) 1653m; decimalLatitude: 43.8773; decimalLongitude: 11.7107; geodeticDatum: WGS84; **Event:** samplingProtocol: Observation; verbatimEventDate: 11.viii.2023
- **Type status:** Other material. **Occurrence:** sex: 1 female; occurrenceID: 6BE9BC53-FEF4-56F3-9D49-4F82CA9F577D; **Taxon:** scientificName: Volucellapellucens (Linnaeus, 1758); order: Diptera; family: Syrphidae; **Location:** country: Italy; locality: PNFC Monte Falco (FI) 1653m; decimalLatitude: 43.8773; decimalLongitude: 11.7107; geodeticDatum: WGS84; **Event:** samplingProtocol: Entomological Net; verbatimEventDate: 11.viii.2023; **Record Level:** collectionID: PNFCC000145

##### Notes

Recorded in Burgio et al. (2000).

#### Volucella zonaria

(Poda, 1761)

BC650AC0-ABEC-5593-8A9B-A9AB3D5D92F0

##### Materials

- **Type status:** Other material. **Occurrence:** sex: 1 male, 1 female; occurrenceID: 03F15E09-D5C4-5D74-8E32-06BE0487E8D3; **Taxon:** scientificName: Volucellazonaria (Poda, 1761); order: Diptera; family: Syrphidae; **Location:** country: Italy; locality: PNFC Bucine (FC) 907m; decimalLatitude: 43.9594; decimalLongitude: 11.7097; geodeticDatum: WGS84; **Event:** samplingProtocol: Entomological Net; verbatimEventDate: 12.viii.2023; **Record Level:** collectionID: PNFCC000146, PNFCC000147
- **Type status:** Other material. **Occurrence:** sex: 1 ex; occurrenceID: CBB39772-E393-552C-87A2-6D3E99546C27; **Taxon:** scientificName: Volucellazonaria (Poda, 1761); order: Diptera; family: Syrphidae; **Location:** country: Italy; locality: PNFC Bucine (FC) 907m; decimalLatitude: 43.9594; decimalLongitude: 11.7097; geodeticDatum: WGS84; **Event:** samplingProtocol: Observation; verbatimEventDate: 12.viii.2023

##### Notes

Recorded in Burgio et al. (2000).

#### Xanthandrus comtus

(Harris, 1780)

C64E7724-C65E-591B-8C98-A3B635D0EFE2

##### Materials

- **Type status:** Other material. **Occurrence:** sex: 1 male; occurrenceID: 99DF4FE1-BD06-589E-BF4F-4372F979ECA9; **Taxon:** scientificName: Xanthandruscomtus (Harris, 1780); order: Diptera; family: Syrphidae; **Location:** country: Italy; locality: PNFC Foresta Lama Fonte Cavalla; decimalLatitude: 43.8238539; decimalLongitude: 11.8835054; geodeticDatum: WGS84; **Event:** samplingProtocol: Malaise Trap; verbatimEventDate: 18.v-8.vi.2004; **Record Level:** collectionID: AMSUB005056
- **Type status:** Other material. **Occurrence:** sex: 1 female; occurrenceID: DD1B6987-5D56-5EB2-9ED2-B89E22FAC25F; **Taxon:** scientificName: Xanthandruscomtus (Harris, 1780); order: Diptera; family: Syrphidae; **Location:** country: Italy; locality: PNFC Foresta Lama Stazione Prati; decimalLatitude: 43.8313707; decimalLongitude: 11.8380049; geodeticDatum: WGS84; **Event:** samplingProtocol: Malaise Trap; verbatimEventDate: 6-16.viii.2003; **Record Level:** collectionID: AMSUB005304

##### Notes

Recorded in Burgio et al. (2000).

#### Xanthogramma pedissequum

(Harris, 1780)

51EE9D61-7FF1-5D1B-A74F-16C0380CBF41

##### Notes

Recorded in Burgio et al. (2000).

#### Xylota segnis

(Linnaeus, 1758)

25804F75-16F4-53AA-9E6E-3552ECEC195B

##### Notes

Recorded in Burgio et al. (2000).

#### Xylota sylvarum

(Linnaeus, 1758)

85D2EDE9-046A-5BB2-91E5-B58ECEED2C02

##### Materials

- **Type status:** Other material. **Occurrence:** sex: 1 male; occurrenceID: DE07ECD7-37A0-50D2-9585-7B4844EF8471; **Taxon:** scientificName: Xylotasylvarum (Linnaeus, 1758); order: Diptera; family: Syrphidae; **Location:** country: Italy; locality: PNFC Foresta Lama P. Saporita; decimalLatitude: 43.821774; decimalLongitude: 11.8802281; geodeticDatum: WGS84; **Event:** samplingProtocol: Malaise Trap; verbatimEventDate: 9.vii.2004; **Record Level:** collectionID: AMSUB005079
- **Type status:** Other material. **Occurrence:** sex: 1 male; occurrenceID: E9B52FAD-5E36-5245-9232-D70381B186AB; **Taxon:** scientificName: Xylotasylvarum (Linnaeus, 1758); order: Diptera; family: Syrphidae; **Location:** country: Italy; locality: PNFC Foresta Lama Stazione Prati; decimalLatitude: 43.8313707; decimalLongitude: 11.8380049; geodeticDatum: WGS84; **Event:** samplingProtocol: Malaise Trap; verbatimEventDate: 6-16.viii.2003; **Record Level:** collectionID: AMSUB005318
- **Type status:** Other material. **Occurrence:** sex: 1 female; occurrenceID: 3B116EAD-36B9-5291-A201-5F29536A9659; **Taxon:** scientificName: Xylotasylvarum (Linnaeus, 1758); order: Diptera; family: Syrphidae; **Location:** country: Italy; locality: PNFC Piedimonte (FI) m. 500; decimalLatitude: 44.0924; decimalLongitude: 11.5185; geodeticDatum: WGS84; **Event:** samplingProtocol: Entomological Net; verbatimEventDate: 6.viii.1994; **Record Level:** collectionID: AMSUB005329

##### Notes

Recorded in Burgio et al. (2000).

#### Xylota tarda

Meigen, 1822

FAF2545A-C151-5F71-8CB1-68E11A09B64D

##### Notes

Recorded in Burgio et al. (2000).

#### Xylota xanthocnema

Collin, 1939

057388EC-8068-5D86-B12F-116CA8514B5C

##### Notes

Recorded in Burgio et al. (2000).

## Discussion

A total of 116 species from FCNP are listed in the present revision, including 25 new recorded species, which considerably update the previous fauna list (Burgio et al. 2000). The new recordings for the FCNP are (in alphabetic order): *Calliceraaurata*, *Cheilosiamelanura*, *Cheilosiaproxima*, *Cheilosiaranunculi*, *Chrysotoxymintermedium*, *Chrysotoxumoctomaculatum*, *Chrysotoxumvernale*, *Dasysyrphusfriuliensis*, *Dasysyrphusvenustus*, *Eriozonasyrphoides*, *Eristalinustaeniops*, *Eristalissimilis*, *Eupeodesbucculatus*, *Eupeodescorollae*, *Heringiaheringi*, *Melangynacompositarum*, *Melanogasternuda*, *Microdonanalis*, *Pipizafestiva*, *Pipizanoctiluca*, *Platycheirusfulviventris*, *Platicheirusimmaculatus*, *Rhyngiarostrata*, *Syrphustorvus* and *Sphaerophorialaurae*. Twelve of these species have been recorded along transects using entomological nets in 2023, twelve using Malaise traps in 2003 and 2004, while four have been collected by G. Campadelli. The fauna of FCNP, reported in this revision, includes several species of conservation interest or whose recording are an updating of Italian Syrphidae regional list in progress (Sommaggio et al., unpublished data). The following species are new records for Emilia-Romagna Region: *Microdonanalis* and *Sphaerophorialaurae*, while *Cheilosiamelanura*, *C.proxima*, *Dasysyrphusfriuliensis*, *Melangynacompositarum* and *Pipizafestiva* are new species records for Toscana Region. Particularly interesting is the presence of a group of species largely distributed in the Alps, but rare or, to date, usually absent from the Apennines. *Sphaerophorialaurae* is the most representative species of this group; this syrphid is known in Europe from Scandinavia and from mountains (in particular Alps and Pyrenees) where it is usually found in alpine grasslands from 2000 m upwards (Speight 2020). In Italy, this species has been recorded only from some localities in the Alps (Romig 2008, Sommaggio 2005, Sommaggio et al. 2023Romig 2008,) . The specimen collected at Stazione Prati (Casa Forestale Lama) by Malaise trap is a particularly interesting record because it is significantly below the altitude where this species is usually found (usually over 1500 m). Other species recorded within FCNP, that are typical of the Alps are:

- *Cheilosiamelanura*: this species is associated with unimproved grassland in mountain areas. In Italy, it has been recorded from the Alps (where it is not rare), while, in the Apennines, it was previously recorded from Lazio Region (Paniccia 2016). In Europe, it is present in the Alps, Carpathians, mountain areas in the Balkans and Caucasus (Speight 2020).

- *Chrysotoxumfasciolatum*: this species is usually associated with open areas in *Fagus*/*Picea* woods. Although it has already been recorded from central Italy (e.g. Birtele et al. (2003), Sommaggio (2005)), this is a rare species south of the Alps.

- *Dasysyrphusfriuliensis*: as the previous species, it is not rare in the Alps, this record in FCNP being the first one south of the Alps.

- *Eriozonasyrphoides*: previously recorded south of the Alps only by Burgio and Daccordi (1997) in a locality of the Apennines (Emilia Romagna Region).

- *Melangynacompositarum*: in Italy, this species has been recorded only in the Alps (Sommaggio 2005), where it is not rare. The European distribution of this Holarctic species is from Fennoscandia to Central Europe and from northern Spain to the Urals; the Alps were previously considered as the southern border of this species (Speight 2020).

- *Platycheirusimmaculatus*: this species has been recorded in Italy only from two localities (one in Trentino Alto Adige Region, the other in Emilia Romagna, unpublished data); it is usually associated with *Fagus*/*Picea* woods and Speight (2020) considers the Alps as the southern border of its distribution in Europe.

- *Eupeodesbucculatus*: this is not a strictly Alpine species; however, most of Italian record of this species are from the North (Sommaggio 2005). It was previously recorded also from peninsular Italy (e.g. Birtele et al. (2003), Paniccia and Toma (2015), Paniccia (2016)), but it is not considered a common species. The record of this species for Sicily (Rondani 1868, Bezzi and De Stefani-Perez 1897) should be confirmed since there is confusion about its identity (Sforzi and Sommaggio 2021).

The particular peculiar climatic conditions in FCNP, with high rainfall, cold winters and cool summers (Padula and Crudele 1988), could create microclimate areas comparable to those of the Alps. In such conditions, northern species could find the climatic condition to survive with residual populations. Viciani et al. (2010) and Viciani and Alberti (2024) underline the presence in FCNP of plants typical of the Alps, even if, from a vegetational point of view, the Park is more similar to other sites in the peninsular Apennines than to Northern Apennines or Alps. Worthy of attention would be to know whether a similar trend could occur for other animal taxa.

Currently, the hoverfly fauna of FCNP (116 species) corresponds approximately to one fifth of Italian fauna (536 species). In the FCNP fauna, 13 species with xylosaprophagous larvae have been recorded, representing 15% of the total Italian saproxylic fauna of hoverflies, although deciduous forests are amongst the most abundant and best-preserved habitats in the Park. It is worth noting that several xylosaprophagous species are elusive and/or early spring species easily recorded with specific sampling (Maritano et al. 2024). Most of the xylosaprophagous species recorded in the FCNP are common species with low ecological requirements, such as, for example, *Xylotasegnis* and *Ferdinandeacuprea*. A few species are uncommon, at least in Italy, such as *Criorhinaasilica*, *Criorhinafloccosa* and *Brachyopapilosa*. Of particular interest are the records in 2023 of two specimens of *C.aurata*. The larvae of this species develop in rot-holes of large, senescent trees of *Fagus*, *Quercus* and *Fraxinus* (Speight 2020). The low development of larvae (2/3 years are necessary to complete the development) and the high ecological requirements make this species particularly vulnerable and considered as threatened in the continent following the recent Red List of European Syrphidae (Vujić et al. 2022). Worthy of mention is also the recording of *Dorosprofuges*, a species associated with old deciduous forests; the larva is probably an ant commensal, likely with *Lasiusfuliginosus* (Latreille) (Speight 2020). Even if recorded from several Italian regions (Sommaggio 2005), this species is considered uncommon in Italy. Three out of the 116 species globally recorded until now in FCNP have been included in threatened categories in the Red List of European Syrphidae (Vujić et al. 2022). In addition to the aforementioned *C.aurata*, it is included within such categories also *Pipizellaelegantissima*, which has been included in the Endangered category by Vujić et al. (2022) mainly based on the very few and scattered known records of this species. To date, *P.elegantissima* has been recorded from a locality in French Alps, from Calabria and from FCNP. Interestingly, this species was recorded in FCNP by Burgio et al. (2000) and it has been confirmed by a recent observation. Little is known about the biology of this species; the larva is probably aphidiphagous like other Pipizinae species, but the prey is unknown (Speight 2020). The species seems to be associated with beech woodlands. It is necessary to increase knowledge on the biology of this species in order to better plan conservation interventions of existing populations. Like *Microdonanalis* (included in the Near Threatened category by Vujić et al. (2022)), also the threatened *M.mutabilis* has myrmecophilous larvae, but the host of this species belongs probably to *Formica* genus and is associated with well-preserved grassland on calcareous substrates.

FCNP has been considered a focus site within “National Biodiversity Future Center” (National Recovery and Resilience Plan, NRRP) and is currently included within the national pollinator monitoring programme (Italy). This Park is a natural area of considerable extension and conservation interest and recordings reported from this revision can be useful for future studies focused on the temporal and spatial trends of biodiversity based on hoverflies. This revision, therefore, constitutes an updating of the hoverfly fauna of Foreste Casentinesi National Park, supplying data for the Italian Regional Checklist that is currently in progress. The present checklist of Syrphidae species in FCNP is surely far from representing the whole hoverfly biodiversity of this wide protected area, because old and recent data only cover a very limited area of the Park. For example, extensive sampling of hoverflies in Dolomiti Bellunesi National Park, using a large sampling effort and multiple sampling techniques, detected more than 200 species (Sommaggio et al. 2023), despite the small extension of the Park (less than half of FCNP). Only a part of the habitats included in FCNP has been sampled for hoverfly fauna; therefore, more research is needed to better understand the biodiversity of this important protected area which, based on the data here reported, shows characteristics of Alpine fauna.
